# Conditional Antimicrobial Peptide Therapeutics

**DOI:** 10.1021/acsnano.2c04162

**Published:** 2022-08-18

**Authors:** Chayanon Ngambenjawong, Leslie W. Chan, Heather E. Fleming, Sangeeta N. Bhatia

**Affiliations:** ^†^Koch Institute for Integrative Cancer Research, ^‡^Institute for Medical Engineering and Science, and ^§^Department of Electrical Engineering and Computer Science, Massachusetts Institute of Technology, Cambridge, Massachusetts 02139, United States; ∥ Howard Hughes Medical Institute, Cambridge, Massachusetts 02139, United States; ⊥ Department of Medicine, Brigham and Women’s Hospital and Harvard Medical School, Boston Massachusetts 02115, United States; # Broad Institute of Massachusetts Institute of Technology and Harvard, Cambridge, Massachusetts 02139, United States

**Keywords:** nanomedicine, conditional therapeutic, antimicrobial peptide, albumin, protease, infection

## Abstract

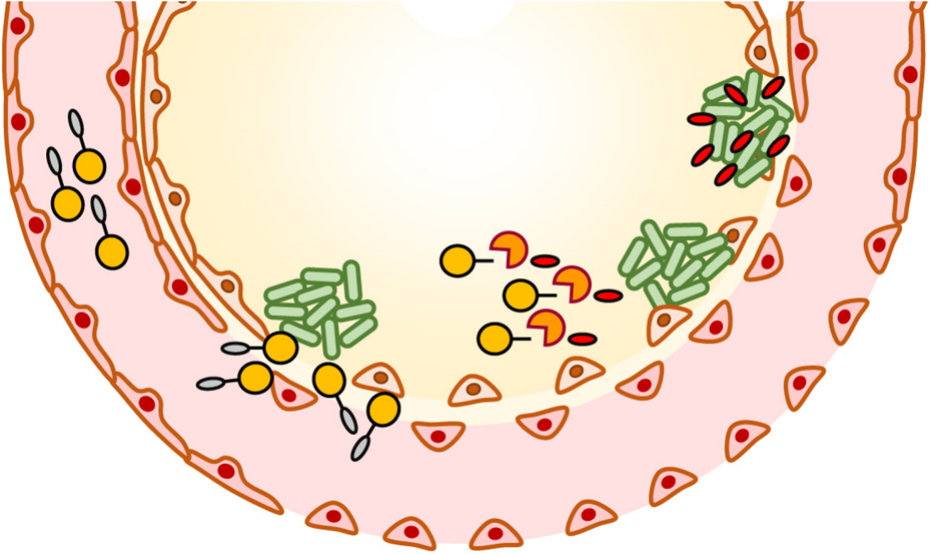

Antimicrobial peptides (AMPs) constitute a promising class of alternatives to antibiotics to curb antimicrobial resistance. Nonetheless, their utility as a systemic agent is hampered by short circulation time and toxicity. Infection sites, analogous to tumors, harbor an aberrant microenvironment that has the potential to be exploited to develop conditionally activated therapeutics with an improved therapeutic index. In particular, we identified strategies to prolong systemic circulation of small, cationic AMPs in a mouse model of bacterial pneumonia. Specifically, we report an albumin-binding domain (ABD)-AMP conjugate as a long-circulating conditional AMP therapeutic with a masked activity that can be liberated by proteases in the infected tissue microenvironment. Our systemically administered conjugate enhanced the pulmonary delivery of active AMP while also reducing AMP exposure to other off-target organs. Importantly, this reduction in off-target exposure improved the safety profile of the AMP. The framework we present can be generalized to quantify and optimize the performance of this emerging class of conditional therapeutics.

Antimicrobial resistance represents a global threat that calls for innovative solutions. Several alternatives to antibiotics are in development at both preclinical and clinical stages.^1,2^ In particular, antimicrobial peptides (AMPs) have garnered attention due to their diverse mechanisms of action ranging from direct bacteria membrane/biofilm disruption to modulation of host immune responses.^3−5^ Nonetheless, clinical translation of AMPs has been sluggish, with colistin being the only cationic AMP approved for parenteral administration to treat multidrug-resistant (MDR) Gram-negative bacteria infection. Most cationic AMPs in clinical trials are formulated for topical administration,^3^ allowing for high drug concentrations to be readily achieved. Major limitations of AMPs that prevent effective intravenous treatment include their high toxicity, poor serum stability, and rapid clearance due to their cationic amphipathic nature and low molecular weight.^4,6^ Additional strategies are needed to optimize and/or reformulate AMPs to enable their successful clinical translation.

Significant progress has been made in terms of AMP discovery and sequence optimization in order to improve clinical translation. Genome mining, machine learning, and structure–activity relationship (SAR) optimization strategies have been employed to derive AMPs with potent activity, improved serum stability, and reduced mammalian toxicity.^7−9^ On the other hand, opportunities exist to leverage diverse drug delivery technologies that have been more conventionally employed by the oncology field to improve the efficacy and therapeutic index of toxic chemotherapeutics or biologics.^10−12^ One notable approach is the development of microenvironment-responsive therapeutics, where active compounds are administered in a pro-formulation and subsequently activated by conditions specific to the tumor microenvironment such as altered proteolysis, acidosis, and hypoxia.^13,14^ Similarly, infection sites also possesses an aberrant microenvironment^15−17^ that can be leveraged for the strategic formulation of infection-responsive pro-therapeutics.^18^

In this work, we report the development of an albumin-binding domain (ABD)-AMP conjugate which, upon association with serum albumin, enables prolonged circulation with a masked antibacterial activity. Importantly, this long-circulating masked AMP can be conditionally activated by protease cleavage of the ABD tether when in an infected organ. We describe a pipeline of *ex vivo* and *in vivo* assays for cleavable linker selection that we created to identify peptide substrates that are readily cleaved in an infected organ with less background cleavage in other nontarget organs. Finally, we comprehensively evaluated the kinetic behavior of our conjugate with respect to organ biodistribution and activation over time. Using a d-stereoisomer version of pexiganan ((d)Pex) as a model AMP in a murine *Pseudomonas aeruginosa* PAO1 lung infection model, we observed that delivery via our ABD-AMP conjugate could yield a higher fraction of active AMP in infected lungs while reducing exposure of the active fraction to off-target organs compared to the free AMP treatment group. The reduction in off-target organ exposure led to an improved safety profile of the AMP. Our initial report on the design and *in vivo* characterization of these ABD-AMP conjugates informs key parameters that favor distribution of active AMP to the target diseased organ after systemic administration and complements the established pool of knowledge in the AMP delivery field to lead to improved understanding and development of protease-activated therapeutics that are not limited to infectious disease applications.

## Results and Discussion

### Design and Synthesis of ABD-AMP Conjugates

**Figure 1:**
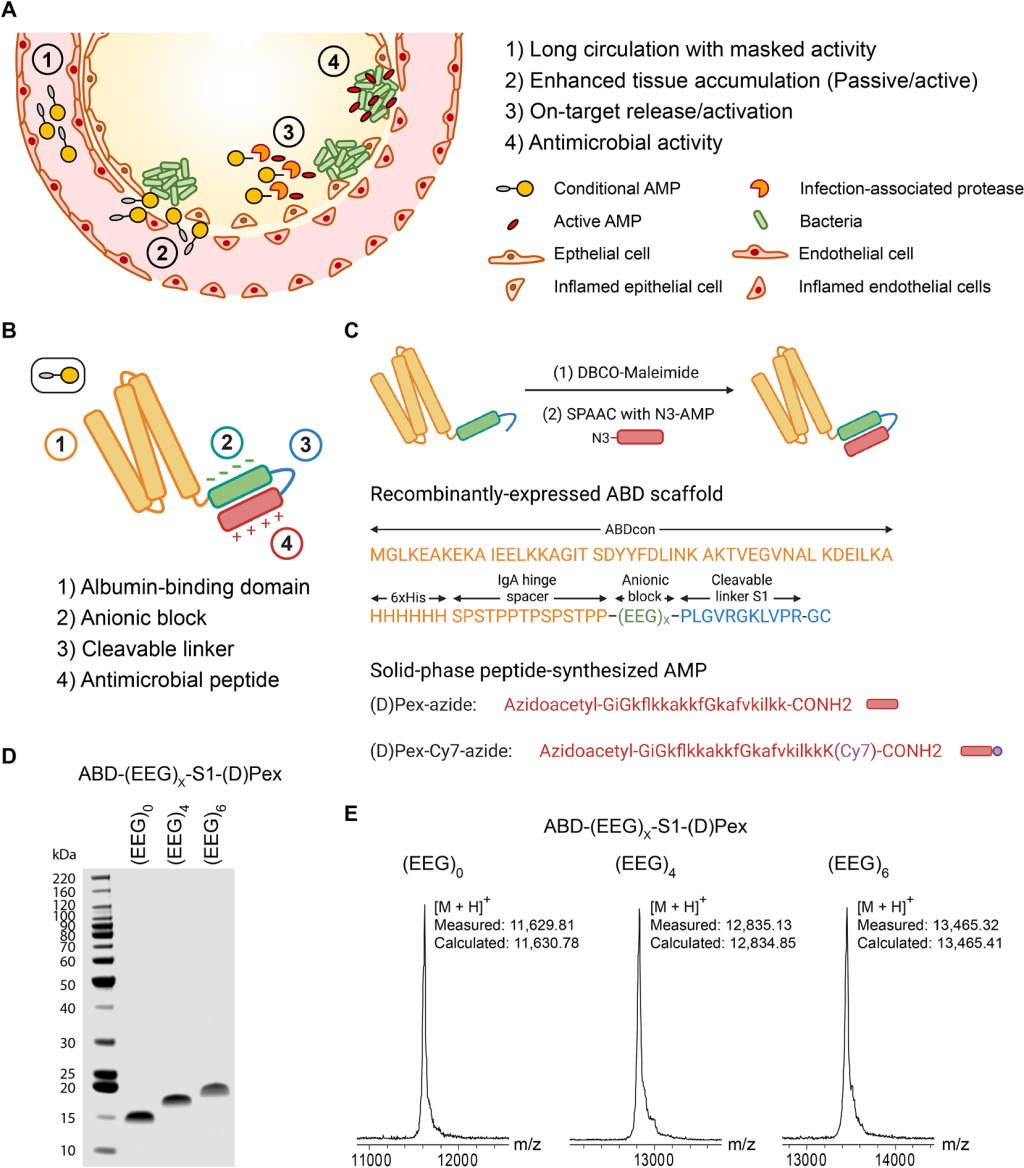
Concept and synthesis of ABD-AMP conjugates. (A) Desirable features of conditional AMP therapeutics include (1) long circulation with masked activity, (2) accumulation at infection site either by passive or active targeting, (3) activation by infection microenvironmental trigger (e.g., infection site proteases), and (4) exhibition of on-target antimicrobial activity. (B) Designed components of ABD-AMP conjugates include (1) albumin-binding domain (ABD), (2) anionic block, (3) cleavable linker, and (4) antimicrobial peptide. (C) Synthesis schematic of ABD-AMP conjugates. ABD-anionic block-cleavable linker-GC was recombinantly expressed in *E. coli.* The C-terminal cysteine was reacted with DBCO-Maleimide cross-linker, followed by strain-promoted alkyne–azide cycloaddition (SPAAC) conjugation with chemically synthesized, azidoacetylated AMP. (D) SDS-PAGE analysis of ABD-AMP conjugates. The conjugates were detected with Coomassie blue staining and imaged on an Odyssey CLx imager. (E) Molecular weights of ABD-AMP conjugates were measured via matrix-assisted laser desorption/ionization-time-of-flight (MALDI-ToF) mass spectrometry (MS) shown as mass-to-charge ratio (*m*/*z*). N-terminal methionine was spontaneously removed during ABD expression.

Major limitations of AMPs during systemic application include their rapid clearance and toxicity to mammalian cells, both of which contribute to their lack of therapeutic efficacy, to date. We envisioned that these challenges could be addressed by formulating a conditional AMP therapeutic that is long-circulating with masked activity, that readily accumulates at the site of infection, and that is subsequently activated in response to infection by disease-associated proteases to enable localized antimicrobial activity (Figure 1A). To achieve these features, we engineered an ABD-AMP conjugate which comprises a concatenation of (1) ABD, (2) anionic block, (3) protease-cleavable linker, and (4) AMP (Figure 1B). Upon hitchhiking to serum albumin via the ABD, the conjugate’s effective size becomes larger than the renal filtration cutoff, thereby reducing renal clearance. In addition, association with albumin also provides steric masking of the AMP activity. To ensure effective activity masking, an anionic block was included in the design to electrostatically complex with AMP cargos, which are typically cationic. Finally, a protease-cleavable linker was inserted between the anionic block and the AMP to enable conditional release of the AMP from the anionic block by active proteases present in the infected microenvironment.

To synthesize the ABD-AMP conjugate, we segmented the construct into two components; (1) the recombinantly expressed “carrier domain” consisting of the ABD, anionic block, and cleavable linker in tandem and (2) the chemically synthesized, azide-functionalized AMP (Figure 1C). A free cysteine was introduced at the C-terminus of the carrier domain to enable site-specific conjugation to the azide-functionalized AMP via a dibenzocyclooctyne (DBCO)-maleimide cross-linker. ABDcon was chosen as our ABD-based albumin binder in this construct due to its high affinity to both mouse and human albumins, as well as the absence of cysteine in the sequence.^19^ Our anionic block was designed as repeats of Glu-Glu-Gly ((EEG)_*x*_) where Glu provides a negative charge and Gly provides flexibility to broadly facilitate complexation with cationic AMPs of diverse sequences and secondary structures. Our initial choice of the cleavable linker (S1) includes a tandem sequence of matrix metalloproteinase (MMP) (PLGVRGK) and thrombin (LVPR)-responsive substrates, considering the biology of *P. aeruginosa* infection which often evokes an injury-associated MMP response as well as thrombosis.^20−22^ The proteolytically stable d-stereoisomer version of pexiganan ((d)Pex) was chosen as a model AMP to represent a cationic, helical AMP (Figure S1). A His6 tag was included as a purification tag due to its small size and low p*K*_a_ of the imidazole side chain (∼6) which should not interfere with albumin-ABD interaction or anionic block-AMP complexation in physiological environment. Each functional element of the conjugate is modular and multiple constructs with different anionic blocks, cleavable linkers, or AMPs can be readily synthesized (Figure 1D,E) to investigate the contribution of each component on the conjugates’ *in vitro* and *in vivo* behaviors.

### ABD-AMP Conjugates with Anionic Block Exhibit Activity Masking with Improved *In Vivo* Lung Accumulation

**Figure 2:**
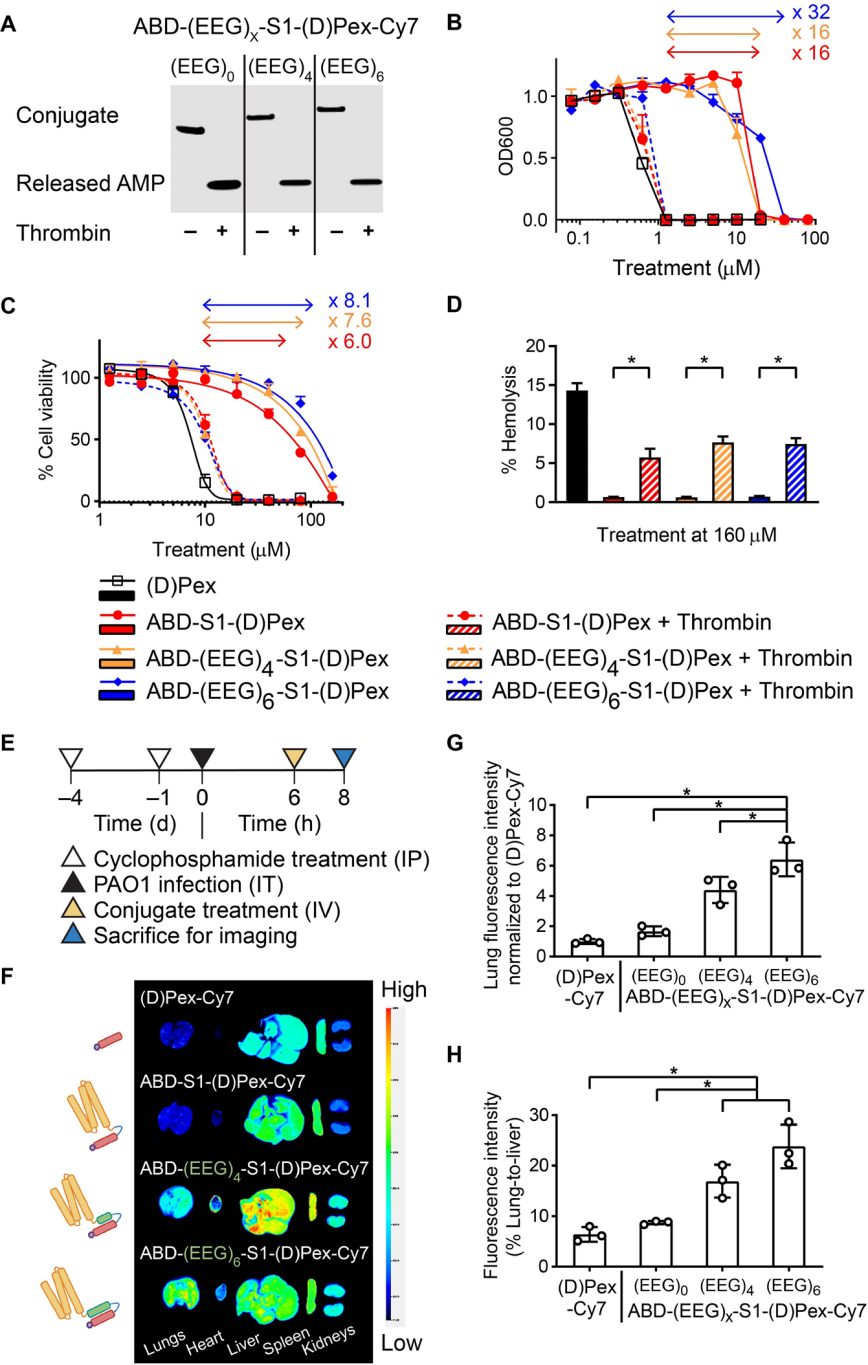
ABD-AMP conjugates with anionic block exhibit activity masking with improved biodistribution. (A) Activation of ABD-AMP conjugates upon incubation with a model protease is shown via SDS-PAGE analysis of ABD-(EEG)_*x*_-S1-(d)Pex-Cy7 without or with thrombin preincubation. The Cy7 signals on intact conjugates and released AMP were detected via an Odyssey CLx imager. (B) Antibacterial activity masking was assessed via microdilution assays on PAO1. Bacteria viabilities following conjugate treatments were measured based on bacterial turbidity at 600 nm (OD600) and normalized to the nontreated control. Fold changes in activity masking were based on the ratios of the minimum inhibitory concentrations (MIC) of the intact and cleaved conjugates. (C) Mammalian toxicity masking was assessed via MTS cell viability assay on L929 fibroblasts. Fold changes in activity masking were based on the ratios of the 50% inhibitory concentrations (IC50) of the intact and cleaved conjugates. (D) Hemolysis masking was assessed via hemolysis assay on mouse red blood cells. (E) Experimental timeline for biodistribution study of ABD-(EEG)_*x*_-S1-(d)Pex-Cy7 conjugates (F) Representative *ex vivo* fluorescence images of conjugate accumulation in different organs imaged on an Odyssey CLx imager (*n* = 3). (G) Quantification of total lung fluorescence signals normalized to (d)Pex-Cy7 control group. (H) Quantification of lung-to-liver fluorescence signals. Panels D, G, and H were plotted as mean ± SD (*n* = 3) and analyzed with One-way ANOVA with Tukey posthoc tests. * denotes statistical significance (*P* < 0.05). IP, intraperitoneal injection; IT, intratracheal instillation; IV, intravenous injection.

To verify protease-dependent activity of ABD-AMP conjugates, we first confirmed their cleavage following incubation with thrombin, a model protease. Three ABD-(EEG)_*x*_-S1-(d)Pex-Cy7 constructs with varying lengths of the anionic block ((EEG)_*x*_) were incubated in thrombin-supplemented phosphate-buffered saline (PBS) for 4 h. Cy7 was attached on the (d)Pex to enable resolution of the intact conjugates from the cleaved AMPs by detecting Cy7 signal following SDS-PAGE analysis of the samples. Indeed, all three conjugates could be readily cleaved by thrombin to release (d)Pex-Cy7 (Figure 2A). Both albumin association and anionic block were found to influence cleavage kinetics of the S1 linker (Figure S2). After confirming cleavability of the conjugates, we next performed microdilution assays in the presence of human serum albumin (HSA) (500 μM) to evaluate antibacterial activity of our conjugates on *P. aeruginosa* PAO1. Steric masking with albumin-associated ABD conferred a 16- to 32-fold change in the minimum inhibitory concentration (MIC) between the intact and cleaved conjugates (Figure 2B, solid versus dotted lines), depending on the length of the anionic block. Specifically, the longest anionic block, (EEG)_6_, improved activity masking by an additional 2-fold (blue lines). Similarly, all masked ABD-AMP conjugates exhibited reduced mammalian toxicity, when tested for killing of L929 fibroblasts (Figure 2C) or hemolysis (Figure 2D). Selectivity index (defined as a ratio of IC50 of intact conjugate on L929 fibroblasts over MIC of activated conjugate on PAO1) was found to increase by at least 9 fold from 5.7 with free (d)Pex to 51.9–64.7 with the conjugates (Table S1). In addition, we observed parallel activity masking outcomes when alternative AMPs, (d)CAMEL0 and Tachyplesin I, were tested in the ABD-(EEG)_6_-S1-AMP formulation (Figure S3). Prior studies to develop pro-AMPs based on anionic masking peptides have been limited to linear AMPs.^23−25^ Here, we demonstrated, with Tachyplesin I as an example, that the anionic peptide block could also be optimized for masking the activity of cyclic AMPs (Figure S3B).

Our goal was to develop ABD-AMP conjugates that would be suitable for systemic application. Hence, we next investigated how our ABD-AMP conjugates are distributed *in vivo* following intravenous administration. To establish a neutropenic mouse model of PAO1 lung infection, cyclophosphamide-treated mice were intratracheally inoculated with PAO1, and 6 h later ABD-(EEG)_*x*_-S1-(d)Pex-Cy7 conjugates with different anionic blocks or free unconjugated (d)Pex-Cy7 were administered via tail vein injection. The mice were euthanized after 2 h and organs were harvested for near-infrared fluorescence imaging with an Odyssey CLx imager (Figure 2E). Despite having a molecular weight below the renal filtration cutoff, (d)Pex-Cy7 was found to accumulate more readily in liver and spleen versus kidneys (Figure 2F). The preferential liver accumulation of some cationic, amphipathic peptides/peptoids was also previously reported, highlighting yet another under-appreciated challenge facing systemic delivery of certain cationic peptides, in addition to renal clearance via kidney filtration.^26−28^ Despite exhibiting antibacterial activity masking *in vitro*, the ABD-S1-(d)Pex-Cy7 conjugate formulated without an anionic block accumulated primarily in liver and spleen, similar to the free peptide control group, implying that the steric masking with serum albumin was not sufficient to mitigate the effect of cationic (d)Pex on liver/spleen accumulation. A similar preferential liver accumulation was also observed in the mice that were administered (d)Pex-conjugated mouse serum albumin (Figure S4). This observation is consistent with a previous study that reported a cationic charge-dependent liver accumulation of cationic small molecule-derivatized albumin.^29^ Encouragingly, when the anionic block was included, we observed relative redistribution of the conjugate accumulation to favor other organs, including the infected lungs (Figure 2F). Specifically, by adding (EEG)_*x*_ block to electrostatically complex the cationic cargo, an approximately 6-fold increase in fluorescence intensity was observed in the lungs of mice treated with ABD-(EEG)_6_-S1-(d)Pex-Cy7 compared to those treated with (d)Pex-Cy7 (Figure 2G). Relative lung-to-liver fluorescence increased from 6.4% in the (d)Pex-Cy7 group to 23.8% in the ABD-(EEG)_6_-S1-(d)Pex-Cy7 group (Figure 2H). Extending the length of the anionic block beyond (EEG)_6_ did not further improve lung accumulation or the ratio of lung-to-liver distribution (Figure S5). To examine the generality of our platform in improving biodistribution of cationic AMPs, we extended our study to test the distribution of (d)KLA (an α-helical AMP) and Tachyplesin I (a β-sheet cyclic AMP) when formulated as an ABD-AMP conjugate (Figure S6). For both cases, the ABD-AMP-Cy7 conjugates with (EEG)_4_ and (EEG)_6_ anionic blocks were found to readily increase lung fluorescence and lung-to-liver distribution relative to their free AMP-Cy7 controls. Thus, our ABD-based carrier with generalized (EEG)_*x*_ anionic block is favorable for activity masking and biodistribution optimization of AMPs.

### Optimization of a Cleavable Linker Increases on-Target Activation in Infected Lungs

**Figure 3:**
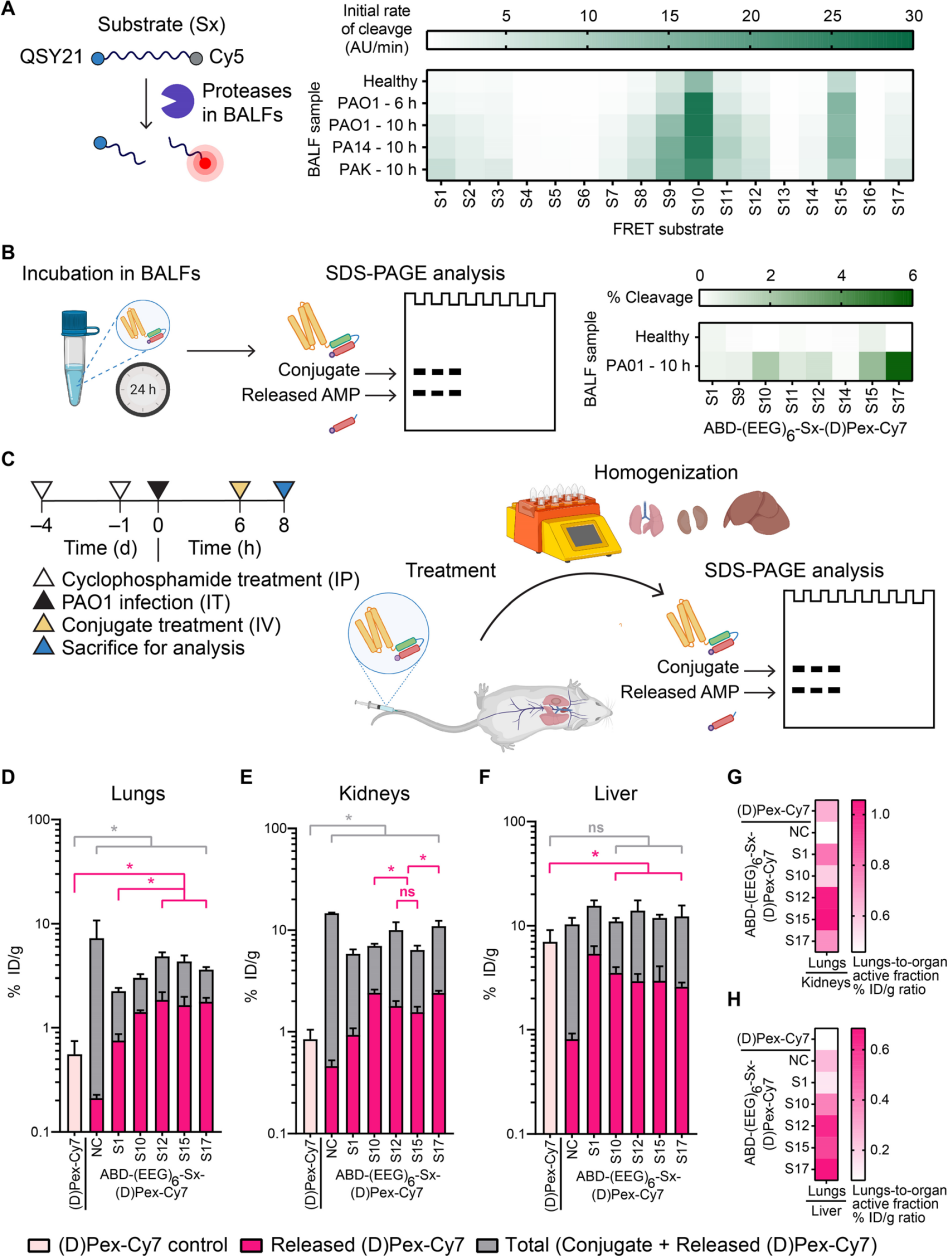
Optimization of cleavable linker improves on-target activation of ABD-AMP conjugate. (A) FRET substrates were screened by incubation with BALFs from healthy and PA-infected mice and monitored for cleavage via Cy5 fluorescence. The average initial cleavage rates of each substrate are represented on the heat map (*n* = 3). (B) ABD-(EEG)_6_-Sx-(d)Pex-Cy7 with different cleavable linkers were screened by incubation in BALFs from healthy and PA-infected mice and analyzed by SDS-PAGE based on Cy7 fluorescence. The average cleavage percentages are represented on the heat map (*n* = 2). (C) Experimental workflow and timeline for *in vivo* evaluation of ABD-AMP conjugate activation. Quantification of total and active fractions of ABD-(EEG)_6_-Sx-(d)Pex-Cy7 in (D) lungs, (E) kidneys, and (F) liver (*n* = 3). Panels D–F were plotted as mean ± SD and analyzed with One-way ANOVA with Tukey posthoc tests. * denotes statistical significance (*P* < 0.05). On-target activation was analyzed based on the lungs/organ % ID/g ratios of each conjugate’s active fraction represented on the heat maps in (G) lungs/kidneys and (H) lungs/liver. Panels B and C were partly created with BioRender.com.

Biodistribution of the active fraction determines the efficacy and toxicity of conditional therapeutics. Therefore, it is desirable that the cleavable linker selected for our ABD-AMP conjugates be preferentially cleaved in the target diseased organ and only minimally released in other organs. We started an initial round of substrate screening with a library of peptide substrates that are responsive to MMPs, thrombin, neutrophil elastase, cathepsins, and PA-secreted proteases (Table S2). Bronchoalveolar lavage fluid samples (BALFs) from noninfected and PA (PAO1, PA14, and PAK)-infected neutropenic mice were used as biofluids representative of lung proteolytic microenvironment for a Förster resonance energy transfer (FRET) cleavage assay. We flanked the peptide substrates with a fluorophore (Cy5)-quencher (QSY21) pair, such that cleavage of the substrates upon incubation with the BALFs could be tracked via a fluorescence signal (Figure 3A). Overall, the BALFs from the PA-infected mice were more proteolytic than those from the healthy mice. Unexpectedly, we did not observe good cleavage signals from substrates S4 and S5 which are reported substrates for PA proteases imelysin and elastase (LasB).^30,31^ In addition, we performed FRET cleavage studies using the PAO1-infected BALFs in the presence of different protease class inhibitors and determined serine proteases and aspartyl proteases to be primary drivers of the cleavage activity observed (Figure S7). From the initial list of substrates, we selected a smaller set of hits and synthesized ABD-AMP conjugates in the ABD-(EEG)_6_-Sx-(d)Pex-Cy7 format to assess cleavage efficiency of the candidate therapeutic constructs (Figure 3B). Cleavage assays of the conjugates were performed by incubation with BALFs, followed by SDS-PAGE analysis to resolve and quantify the amount of the intact conjugate from the cleaved (d)Pex-Cy7. We observed some differences in relative cleavage efficiency between the FRET substrates and the ABD-AMP conjugates. Specifically, substrate S10 exhibited higher cleavage rates than substrate S15 in the FRET format, whereas the trend was opposite in the conjugate format where ABD-(EEG)_6_-S15-(d)Pex-Cy7 was more efficiently cleaved. This difference is possibly due to additional factors such as higher steric hindrance or a more highly charged environment near the cleavage site of the conjugate, thus emphasizing the importance of validating cleavage efficiency in the actual therapeutic construct. Nonetheless, the FRET assay serves as a valuable higher-throughput first-pass screen to narrow down the list of potential hit substrates for subsequent validation in the therapeutic conjugate format.

Next, we selected the four hit substrates (S10, S12, S15, and S17) from the *ex vivo* conjugate cleavage assay for evaluation of *in vivo* activation in a PAO1 lung infection model. The conjugate with substrate S1, which was our initial substrate choice, as well as the noncleavable (NC) control were also included in this study for comparison. Six hours after intratracheal instillation of PAO1, ABD-(EEG)_6_-Sx-(d)Pex-Cy7 with different linker substrates and (d)Pex-Cy7 control were intravenously administered and the mice were euthanized 2 h later (Figure 3C). Subsequently, lungs, liver, and kidneys were homogenized and supernatants were collected for SDS-PAGE analysis. When quantified in terms of percentage of injected dose/gram (% ID/g), the NC control had the least activated fraction, confirming the necessity of a cleavable linker to enable payload release (Figure 3D). Among the cleavable conjugates, the S12, S15, and S17 conjugates yielded the highest % ID/g of released (d)Pex-Cy7 in the lungs. When analyzing the released AMP fraction in kidneys, the S12 and S15 conjugates led to lower % ID/g than the S17 conjugate and are therefore preferred (Figure 3E). Favorably, when looking at the liver, the primary sequestration organ of (d)Pex-Cy7, we observed the lowest levels of the active fraction when S12, S15, and S17 conjugates were used, compared to the free (d)Pex-Cy7 control group (Figure 3F). Thus, S12 and S15 conjugates represent our two best hits in terms of higher released AMP % ID/g in the lungs, paired with lower released AMP % ID/g in liver and kidneys, also illustrated by their higher % ID/g ratios when comparing the active fractions in lungs versus off-target organs (Figure 3G, kidney and Figure 3H, liver). It is worth noting that substrate S12 was not the best hit in either *ex vivo* FRET or conjugate cleavage assays, thus highlighting additional complicating factors in the *in vivo* environment, such as the lung-specific physiological milieu, which could differ from PBS-flushed BALF samples used in the *ex vivo* assays. Therefore, the *in vivo* study of conjugate activation provides valuable information in determining the best conjugate that is more preferentially activated in the diseased organ (infected lungs) over the other clearance organs (liver and kidneys), which is a crucial trait in maximizing efficacy and therapeutic index of conditional therapeutics. Our current *ex vivo* substrate screening was based solely on the substrates that were efficiently cleaved in infected BALFs (on-target activation). In the future, it should be possible to expand our *ex vivo* substrate screening and also include screening against kidney/liver homogenates (off-target activation) to further inform substrates that are likely to be more preferentially activated in the infected lungs with less off-target activation for *in vivo* evaluation.

### Optimized ABD-AMP Conjugate Exhibits Time-Dependent Activation with High Infected Lung-to-off-Target Organ Selectivity

**Figure 4:**
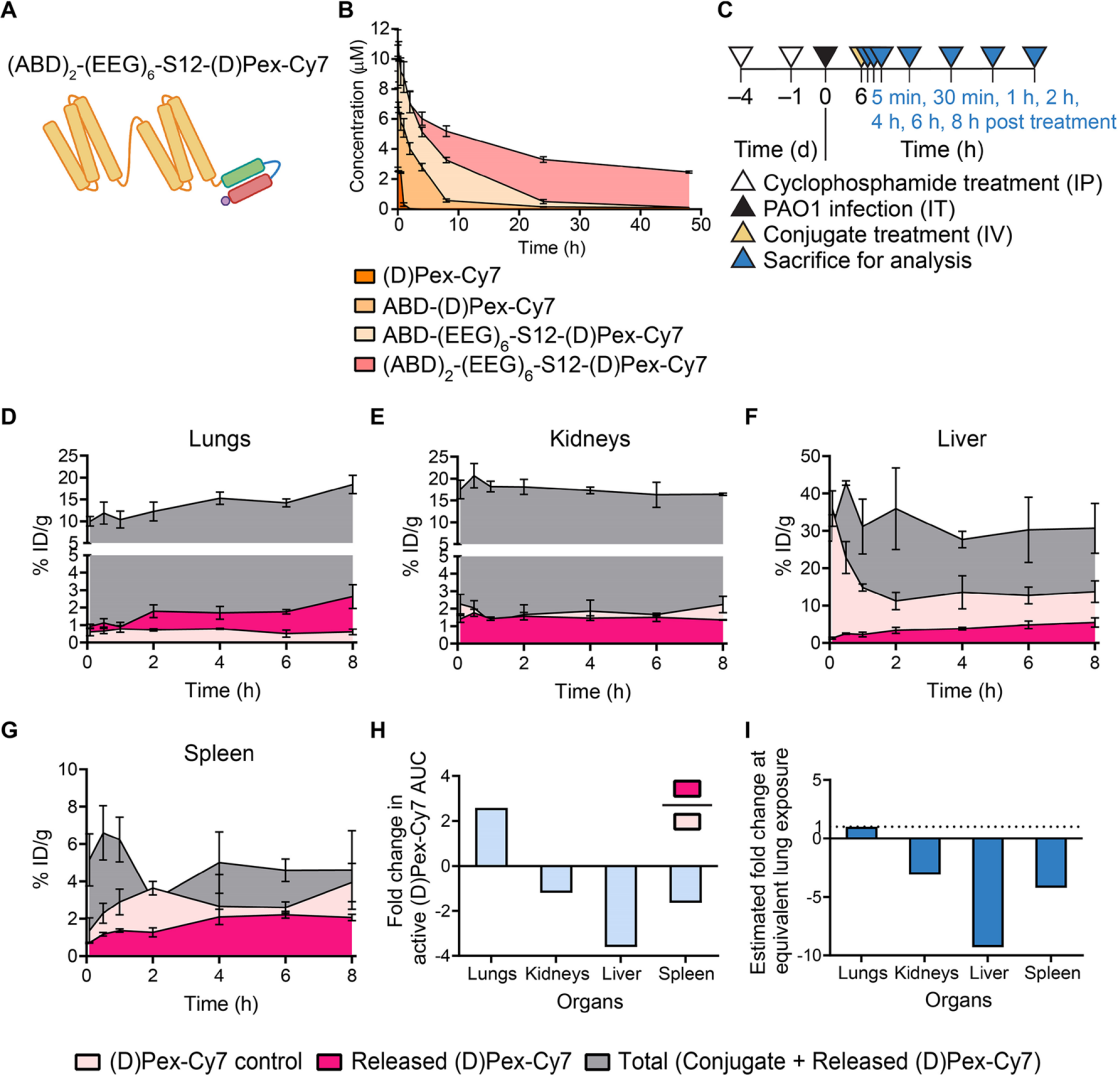
Optimized ABD-AMP conjugate improves circulation time and longitudinal on-target accumulation of active AMP. (A) Schematic of divalent ABD-AMP conjugate. (B) Pharmacokinetics of ABD-AMP conjugates. (*n* = 4). (C) Experimental timeline for longitudinal biodistribution study. Accumulation over time of (ABD)_2_-(EEG)_6_-S12-(d)Pex-Cy7 (released and total fractions) and (d)Pex-Cy7 in (D) lungs, (E) kidneys, (F) liver, and (G) spleen. (*n* = 3–4). (D–G) were plotted as mean % ID/g ± SD (H) Fold change in accumulation (AUC) of the released (d)Pex-Cy7 fraction of the conjugate relative to the free (d)Pex-Cy7 control in different organs. Reduced accumulations relative to the control were plotted as negative fold change. (I) Estimated fold change in accumulation (AUC) of the released (d)Pex-Cy7 fraction of the conjugate relative to the free (d)Pex-Cy7 control in off-target organs at equivalent lung exposure.

Central to the conditional therapeutic concept is preferential activation at the diseased site with minimized off-target exposure. However, to our knowledge no known study to date has comprehensively evaluated the multiorgan kinetics of conditional AMP therapeutic accumulation and activation to quantitatively demonstrate whether the desirable shift in active compound exposure was indeed achieved *in vivo*. Hence, we set out to evaluate our ABD-AMP conjugate *in vivo* with regard to biodistribution and extent of activation over time in different organs. With our optimized cleavable linker, we focused our study on divalent (ABD)_2_-(EEG)_6_-S12-(d)Pex-Cy7 (Figures 4A, S8, and S9) which is our most long-circulating construct (Figures 4B and S10, and Table S3) with enhanced activity masking (Figure S11 and Table S1). The conjugate exhibited similar *in vitro* cleavage kinetics as the monovalent conjugate (Figure S12) but was found to better deliver active AMP to PAO1-infected lungs compared to both monovalent and nonalbumin-binding conjugates (Figures S13 and S14). In the PAO1 lung infection model, (ABD)_2_-(EEG)_6_-S12-(d)Pex-Cy7 and (d)Pex-Cy7 control were administered to the infected mice at 6 h post infection (Figure 4C). After different time points up to 8 h post treatment, cohorts of 3–4 mice were euthanized and organs were harvested for quantification of the total conjugate versus released (d)Pex-Cy7. In the infected lungs, we observed an increase in both total and active fractions of the conjugate over time, while the amount of (d)Pex-Cy7 in the free AMP control group remained steady for the first 4 h before gradually declining (Figure 4D). Over the 8 h time frame, we did not observe a complete activation of the conjugate, pointing out room for further optimization of the cleavable linker. The increase in the released AMP of the conjugate over time was confirmed to be driven by infection in the lungs, as the released AMP content remained steady at the 5 min time point level throughout the course of study in the lungs of the noninfected mice (Figure S15). In addition, infection-dependent preferential activation of the conjugate was also observed in other lung infection models using different PA strains including clinical isolates (Figure S16) as well as in a PAO1 thigh infection model (Figure S17). In kidneys, liver, and spleen, the amount of released (d)Pex-Cy7 remained below that of the free AMP control throughout the time-course of study (Figure 4E–G). When quantified using an area under the concentration–time curve (AUC) analysis, (ABD)_2_-(EEG)_6_-S12-(d)Pex-Cy7 delivered 2.6-fold more active (d)Pex-Cy7 to the infected lungs compared to the free (d)Pex-Cy7 control, while also reducing free AMP exposure to kidneys, liver, and spleen by 1.2-, 3.6-, and 1.6-fold, respectively (Figure 4H and Table S4). At the equivalent levels of exposure to active (d)Pex-Cy7 in the infected lungs, the conjugate is estimated to reduce off-target exposure to the active AMP by 3.1, 9.3, and 4.2 fold in kidneys, liver, and spleen, respectively (Figure 4I). This estimation was further confirmed by a follow-up dose-varying biodistribution study, such that doses of 5, 15, and 45 nmol were administered, which allows us to directly compare the levels of active AMP present in different organs at different doses (Figure S18). Of note, the degree of reduction in off-target exposure is dependent on the inherent biodistribution of the formulated AMP, given that when we conducted the biodistribution study using another model AMP (d)KLA, we observed that the reduction in kidney exposure to active AMP was even more pronounced than what was detected in the (d)Pex conjugate study, due to a higher inherent accumulation of (d)KLA in kidneys (Figures S6 and S19). Taken together, our ABD-AMP formulation is a promising conditional AMP platform that can effectively redistribute active AMP toward organ of interest (infected lungs) away from off-target organs following systemic administration. It is worth noting that we employed serum-stable AMPs (either d-stereoisomers or cyclic AMPs) in our current studies and hence, the accumulated amounts could be less for other AMPs that are susceptible to serum degradation. We envision that selection and/or optimization of proteolytically stable AMPs would be essential in complementing our ABD-AMP formulation.

### Optimized ABD-AMP Conjugate Improves Safety Profile of AMP

**Figure 5:**
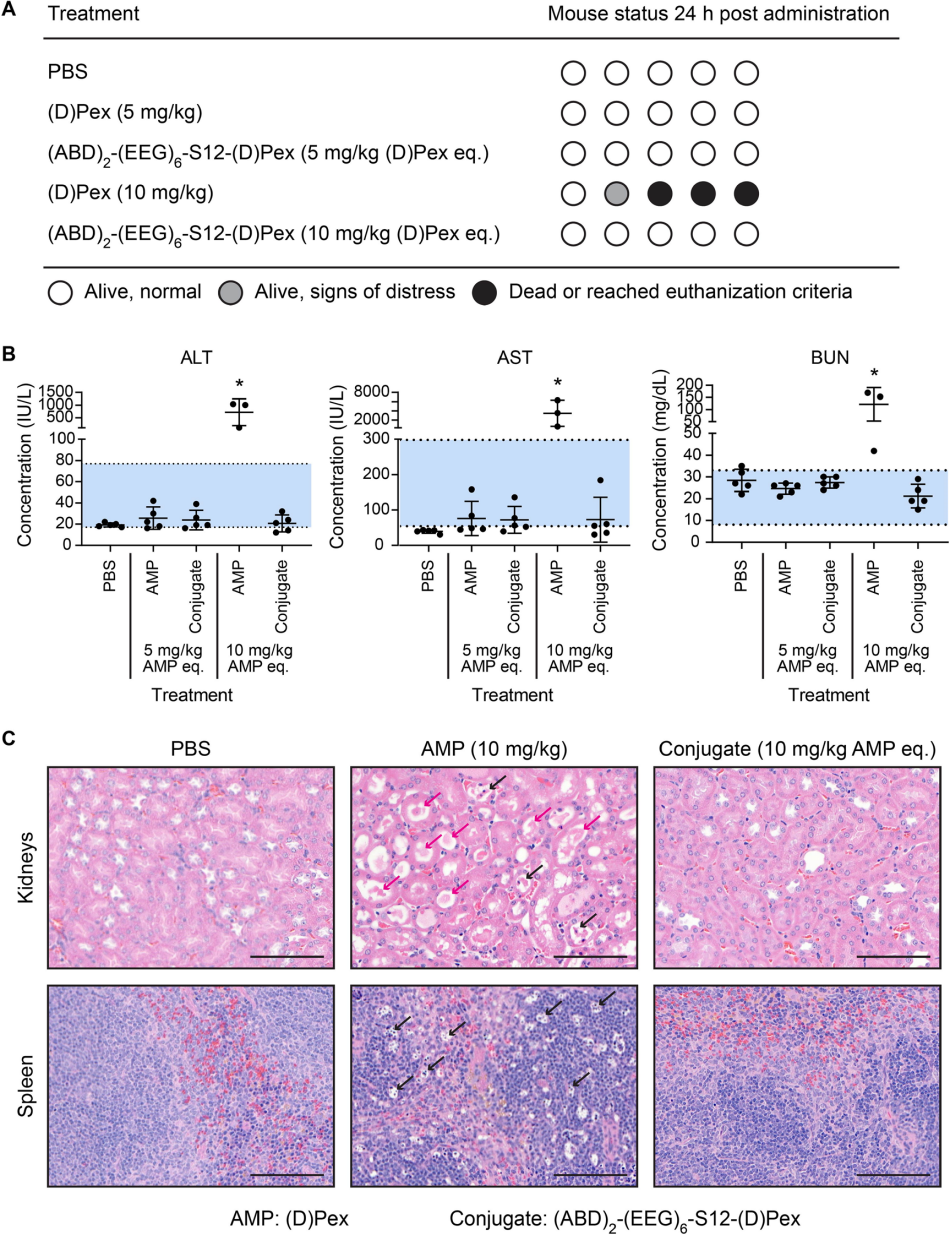
Optimized ABD-AMP conjugate exhibits enhanced safety profile. (A) Treatment groups and status of the treated mice. Each circle represents a mouse. (B) Serum chemistry analysis of the mice in different treatment groups. The serum levels are shown for ALT, AST, and BUN plotted as mean ± SD and analyzed with One-way ANOVA with Tukey posthoc tests. * denotes statistical significance (*P* < 0.05). (*n* = 3–5). Blue area indicates a normal reference range. The serum levels of additional analytes are reported in Figure S20. (C) Histological evaluation of organ sections (kidneys and spleen) stained with H&E (*n* = 3). Scale bar represents 100 μm. Magenta arrows indicate protein casts. Black arrows indicate apoptotic cells. The sections of the other organs (heart, lungs, and liver) are shown in Figure S22.

Systemic toxicity poses a critical challenge to clinical translation of AMP therapeutics. Nephrotoxicity is a well-documented side effect of polymyxins used in clinics.^32^ In addition, mammalian cell toxicity and hemolysis were also observed in several preclinical AMPs.^33^ In order to improve therapeutic index of AMPs, it is important that the AMPs be modified or formulated to reduce off-target toxicity. Given favorable *in vitro* toxicity masking and reduction in off-target organ exposure of the systemically administered ABD-AMP conjugate, we next evaluated whether this shift in biodistribution could lead to an improved safety profile of the formulated AMP. Mice were intravenously administered (d)Pex or (ABD)_2_-(EEG)_6_-S12-(d)Pex at 5 and 10 mg/kg AMP eq and were monitored for 24 h. At the end point, the mice were euthanized and serum and organs were collected for serum chemistry analysis and histology evaluation, respectively. Over the observation period, signs of distress were only observed in the mice treated with (d)Pex at 10 mg/kg and 3 of 5 mice in this group did not survive to the study end point (Figure 5A). In contrast, the mice that were treated with the conjugate at 10 mg/kg AMP eq exhibited no sign of distress. Maximum tolerated dose (MTD) of (d)Pex was determined to be 5 mg/kg whereas MTD of (ABD)_2_-(EEG)_6_-S12-(d)Pex was not reached at the highest dose tested (10 mg/kg AMP eq) which is near the solubility limit of the conjugate. Serum levels of alanine aminotransferase (ALT), aspartate aminotransferase (AST), blood nitrogen urea (BUN), and creatinine were elevated beyond a normal reference range in the mice treated with 10 mg/kg (d)Pex, indicating liver and kidney dysfunction in this treatment group (Figures 5B and S20A). The levels of these analytes remained within the reference range for the other treatment groups. Alkaline phosphatase and serum albumin levels were not statistically altered in any of the treatment groups compared to the PBS control group (Figure S20B,C) and mouse body weight remained higher than 95% of the starting weight for all treatment groups (Figure S20D). Pro-inflammatory cytokine IL-6 was found to be significantly elevated only in the serum samples of the mice treated with 10 mg/kg (d)Pex (Figure S21). With regard to histology, the harvested organs were fixed, sectioned, stained with hematoxylin and eosin (H&E), and evaluated by a veterinary pathologist blind to the treatment groups. Pathological damage was observed in kidneys and spleen of the mice treated with 10 mg/kg (d)Pex. Specifically, in this treatment group, dilated renal tubules with protein casts and apoptotic epithelial cells were observed in the kidney sections, and patches of apoptotic lymphocytes were observed in the spleen sections (Figure 5C). On the other hand, treatment with (ABD)_2_-(EEG)_6_-S12-(d)Pex at 10 mg/kg AMP eq did not result in any observable histological damage in any evaluated organs (Figures 5C and S22). We did not observe abnormalities in the liver sections of the (d)Pex (10 mg/kg) group despite high serum levels of ALT and AST (Figure S22). To ensure broad applicability of the platform, improved tolerability of AMP with the ABD-AMP formulation was demonstrated in another model AMP (d)KLA (Figure S23). Altogether, we showed that ABD-AMP conjugate formulation improves the safety profile of AMPs, which, together with enhanced bioavailability at the diseased site, supports further investigation of the conjugate as a systemic conditional antimicrobial agent.

## Conclusions

We report the development of a long-circulating ABD-AMP conjugate that enhances active AMP accumulation in the infected organ while lowering its exposure in the other organs. The modulation in biodistribution, together with the masked mammalian toxicity and hemolysis, improved safety profiles of the formulated AMPs. Future iterations of this conjugate could incorporate active-targeting domains to promote target organ accumulation/bacteria engagement and could be further refined in terms of the selection of more efficient/specific cleavable linkers (either disease or organ-specific). Our effort in conditional AMP formulation would go hand in hand with efforts from other groups focused on the discovery and optimization of potent AMPs^34−38^ as well as exploration of AMP/AMP or AMP/antibiotic synergistic combinations,^39−41^ all of which would help accelerate clinical translation of AMPs for systemic administration. Extending beyond a model AMP (d)Pex, future exploration on different AMP candidates will take into account their potential immunomodulation and antibiofilm properties which are increasingly recognized as important attributes to improve antibacterial efficacy.^42−45^ Given that the currently used acute infection mouse model develops more rapidly than the course of acute infection in humans, future work will look into evaluation of our conjugate in a chronic infection model that might provide a more relevant context for therapeutic efficacy assessment of our pro-therapeutic which takes longer than free AMP to reach maximal concentration. As a first step, our current contribution delineates features that improve *in vivo* characteristics and safety profiles of the conditional AMP therapeutic supporting its future optimization and investigation for clinical translation. Although our current investigation was focused on lung infection models, our systemic formulation should be applicable to other infection contexts where topical administration of therapeutic is not viable. Additionally, several cationic, amphipathic AMPs are also regarded as anticancer peptides due to their preferential cytolytic activity on cancer cells, which possess more negatively charged cell membrane than that of normal cells.^46^ We envision that our conjugate formulation could be tailored to more selectively deliver these anticancer peptides to tumors for oncology applications. More broadly, our platform could be adapted for enhanced on-target delivery of peptide therapeutics in various diseases with aberrant microenvironments. The developmental framework may be useful in guiding optimization of other conditional/stimuli-responsive therapeutics with alternative carrier proteins or polymers.

## Methods

### Molecular Cloning of ABD Backbone

Double-stranded DNA gBlocks gene fragments encoding ABD, anionic block, cleavable linker, and C-terminal cysteine with flanking NcoI and XhoI restriction sites were ordered from Integrated DNA Technologies (IA, U.S.A.). The gene fragments were cloned into Novogen pET28a(+) vector at the NcoI and XhoI restriction sites and transformed into DH5α competent *E. coli* cells (New England Biolabs Inc., MA, U.S.A.). Selection of the correctly cloned bacterial colonies was confirmed by Sanger sequencing (Quintara Biosciences, CA, U.S.A.). The corresponding plasmids were harvested and transformed into BL21(DE3) competent *E. coli* cells (New England Biolabs Inc., MA, U.S.A.) for protein expression.

### Recombinant Expression of ABD Backbone

A secondary culture of BL21(DE3) *E. coli* encoding ABD backbone (500 mL in LB broth supplemented with 50 μg/mL of kanamycin) was grown at 37 °C from an overnight primary culture (3 mL) until optical density at 600 nm (OD600) reached about 0.6–0.8. Protein expression was induced with an addition of isopropyl β-d-1-thiogalactopyranoside (IPTG) (1 mM final concentration) followed by a 2 h incubation at 37 °C. The bacteria were then pelleted and stored in a −80 °C freezer. For purification, the bacteria pellet was first thawed on a 37 °C water bath, lyzed with B-PER complete bacteria protein extraction reagent (ThermoFisher Scientific, MA, U.S.A.) and centrifuged at 11 000 rpm for 20 min. ABD in the clarified supernatant was purified via a standard immobilized metal affinity chromatography (IMAC) with Ni-NTA agarose (Qiagen, MD, U.S.A.) in Tris buffer. The product was confirmed via SDS-PAGE analysis.

### Synthesis of ABD-AMP Conjugates

All AMPs (Table S5) were synthesized via a standard Fmoc solid-phase peptide synthesis from CPC Scientific (CA, U.S.A.) with at least 95% purity (Figure S24). ABD was first incubated with tris(2-carboxyethyl)phosphine (TCEP) (20 equiv) for 1 h at room temperature (RT) to reduce its C-terminal cysteine. The reduced protein solution was then centrifuge-filtered with a 10-kDa Amicon centrifugal filter unit (MilliporeSigma, MA, U.S.A.) (4 times) to remove free TCEP and exchange its buffer into PBS (pH 6.5, 1 mM EDTA). Subsequently, the reduced protein was reacted with DBCO-Maleimide cross-linker (Click Chemistry Tools, AZ, U.S.A.) (5 equiv) for 5 h at RT. The DBCO-functionalized ABD was purified with a disposable PD-10 desalting column (GE Healthcare Bio-Sciences, PA, U.S.A.) to remove unreacted cross-linker and exchange its buffer into PBS (pH 7.4). AMP conjugation step was performed on solid phase support as follows. First, the DBCO-functionalized ABD was preadsorbed onto Ni-NTA agarose for 15 min after which a stock solution of AMP in water (1.5 equiv) was added to the mixture. The reaction was incubated overnight at RT. Next, excess AMP was extensively washed off with Tris buffer (pH 8) and the product was eluted with Tris buffer (500 mM imidazole, pH 8). Finally, the product was buffer-exchanged into PBS (pH 7.4) with a disposable PD-10 desalting column and concentrated with a 10-kDa Amicon centrifugal filter unit. All synthesized conjugates were confirmed via SDS-PAGE analysis and quantified by either 220 nm absorption for unlabeled conjugates or 740 nm absorption for Cy7-labeled conjugates. Molecular weights of the conjugates were confirmed via MALDI-ToF MS (Bruker Autoflex) using α-cyano-4-hydroxycinnamic acid as a matrix (Table S6). Lead conjugates were additionally characterized via analytical high pressure liquid chromatography (HPLC) using a C4 analytical HPLC column (Figure S25).

### Microdilution Assay

*P. aeruginosa* PAO1 was a generous gift from the Ribbeck Lab at the Massachusetts Institute of Technology. ABD-AMP conjugates were 2-fold serially diluted in MHB media on 96-well plates with triplicates per treatment. For assessment of the cleaved conjugates, the conjugates were incubated with human thrombin (Haematologic Technologies, VT, U.S.A.) (125 nM) for 4 h prior to the serial dilutions. An equal volume of PAO1 suspension in HSA-supplemented MHB was added to the 96-well plates to achieve a final concentration of 5 × 10^5^ cfu/mL PAO1 in 500 μM HSA for each treatment well. The plates were incubated at 37 °C for 16 h after which OD600 was measured by Infinite 200 PRO plate reader (Tecan, Switzerland) to determine MIC.

### Mammalian Toxicity Assay

L929 fibroblasts were seeded on tissue culture-treated 96-well plates at 10 000 cells/well in Dulbecco’s modified Eagle medium (DMEM) media (10% fetal bovine serum, 1% penicillin-streptomycin solution) and cultured in an incubator at 37 °C, 5% CO_2_. After 24 h, the culture media was replaced with treatment media containing 2-fold serially diluted ABD-AMP conjugate solutions in DMEM (500 μM HSA) and incubated for 4 h. Cell viability was measured after 24 h using MTS-based CellTiter 96 aqueous one solution cell proliferation assay (Promega, WI, U.S.A.).

### Hemolysis Assay

Fresh blood was collected from a CD-1 mouse via cardiac puncture using a syringe precoated with 0.1 mL EDTA solution (50 mM). Red blood cells were separated from plasma components via centrifugation and further washed with 150 mM sodium chloride solution. The final red blood cell suspension was added to 96-well plates and treated with 2-fold serially diluted ABD-AMP conjugate solutions for 1 h at 37 °C. Then, red blood cells were pelleted and supernatants were transferred to new 96-well plates for measurement of 541 nm absorbance to quantify released hemoglobin. Triton-X 100 treatment (0.1%) was used as a positive control to normalize percent hemolysis.

### Mouse Model of PAO1 Lung Infection and BALF Collection

All animal studies were approved by the Massachusetts Institute of Technology’s Committee on Animal Care (MIT protocol 0619-034-22). CD-1 mice (11–12 weeks old) were rendered neutropenic by intraperitoneal injections of cyclophosphamide at 4 d (150 mg/kg) and 1 d (100 mg/kg) prior to infection. PAO1 inoculation was performed by intratracheal instillation of PAO1 suspension (2 × 10^5^ cfu in 50 μL PBS) via a 22G blunt-end catheter (EXCEL International). For BALF collection, the mice were first euthanized. An incision into the trachea was made with a needle puncture where lavage was performed with PBS (1 mL) via a catheter-fitted syringe. BALFs were centrifuged to remove cells, aliquoted, and stored in a −20 °C freezer.

### BALF Cleavage Assay

FRET peptide substrates in PBS (15 μL, 40 μM) as listed in Table S2 were incubated with an equal volume of BALFs in a 384-well plate. Fluorescence signal of Cy5 was monitored for 2 h by a plate reader. Cleavage rate of each substrate was calculated from the initial slope of the Cy5 signal. Cleavage assay on ABD-AMP-Cy7 conjugates was performed by incubation of the conjugate solutions (15 μL, 20 μM) with an equal volume of BALFs in the presence of HSA (500 μM) for 24 h followed by SDS-PAGE analysis using NuPAGE 4 to 12% Bis-Tris protein gels in a MES running buffer. Signals of the intact conjugates and cleaved AMPs were measured using an Odyssey CLx imager at the 800 nm channel (LI-COR Biosciences, NE, U.S.A.) and calculated for percent cleavage.

### Biodistribution Study

ABD-AMP-Cy7 conjugates (15 nmol) were intravenously administered at 6 h post infection. The mice were euthanized 2 h after and organs were harvested for imaging with an Odyssey CLx imager. After imaging, organs were homogenized in PBS supplemented with Halt protease inhibitor cocktail (ThermoFisher Scientific, MA, U.S.A.) using a gentleMAC dissociator (Miltenyi Biotec, CA, U.S.A.). The homogenates were pelleted and the supernatants were used for SDS-PAGE analysis.

### Pharmacokinetics Study

ABD-AMP-Cy7 conjugates (15 nmol) were intravenously administered to CD-1 mice. At different time intervals, blood samples (5 μL) were collected via saphenous vein bleeding using heparin-coated capillary tubes. Blood samples were diluted 10-fold in PBS supplemented with Halt protease inhibitor cocktail and EDTA and centrifuged to remove cells. The supernatants were used to quantify conjugate concentration via SDS-PAGE analysis.

### Toxicity Evaluation

ABD-AMP conjugates at different doses were intravenously administered to CD-1 mice. The mice were closely monitored for 24 h before euthanization unless significant morbidity was observed that necessitated early euthanization. Blood samples were drawn via cardiac puncture and dispensed into serum separator tubes (BD Microtainer). The samples were left to clot for at least 30 min and spun at 15 000 rcf for 1.5 min to separate serum. The serum samples were frozen in a −20 °C freezer and submitted for serum chemistry analysis by MIT Diagnostic Laboratory (MIT Division of Comparative Medicine). Serum levels of pro-inflammatory cytokine IL-6 and TNF-α were quantified using ELISA MAX standard sets (BioLegend, CA, U.S.A.) following the manufacturer’s protocol. Organs were harvested, fixed in 10% formalin at RT for 24 h, sectioned into 5 μm slices, and stained with H&E for analysis. The slides were evaluated by a veterinary pathologist blind to the treatment groups and were digitized using a Pannoramic digital slide scanner at 20× magnification.

### Statistical Analysis and Schematic Representation

All statistical analysis was performed on GraphPad Prism software (GraphPad Software Inc., CA, U.S.A.). Data were plotted as mean ± standard deviation. Comparison among different treatment groups was based on One-way ANOVA with Tukey posthoc tests. *P* < 0.05 was considered statistical significance. Schematics were partly created with BioRender.com (Toronto, Canada).

## Supporting Information Available

Supplementary methods, Figure S1; stability of (l)Pex and (d)Pex in thrombin, Figure S2; albumin association and anionic block affect cleavage kinetics of ABD-S1-AMP conjugates by thrombin and mouse serum, Figure S3; ABD-AMP conjugates exhibit activity masking across multiple model AMPs, Figure S4; conjugation of (d)Pex to MSA increased liver accumulation, Figure S5; improvement in biodistribution of ABD-AMP conjugate is saturated after reaching the threshold anionic block length, Figure S6; ABD-AMP conjugates with anionic block improves biodistribution of multiple model AMPs, Figure S7; proteolytic activities of BALFs on FRET substrates were inhibited by multiple class-specific protease inhibitors, Figure S8; schematic and characterization of divalent ABD-AMP conjugate, Figure S9; characterization of albumin binding by ABD and ABD-AMP conjugates, Figure S10; (ZHER2)_2_-(EEG)_6_-S12-(d)Pex-Cy7, as a nonalbumin-binding control conjugate with anionic block, exhibits short circulation time, Figure S11; divalent ABD-AMP conjugate exhibits enhanced activity masking, Figure S12; divalent association with two albumins did not affect cleavage kinetics of (ABD)_2_-(EEG)_6_-S12-(d)Pex-Cy7, Figure S13; divalent ABD-AMP conjugate delivers more active AMP to bacteria-infected lungs, Figure S14; albumin association of ABD-AMP conjugate is necessary for enhanced delivery of active AMP to bacteria-infected lungs, Figure S15; The level of active AMP remains steady in the noninfected lungs, Figure S16; ABD-AMP conjugate is preferentially activated in bacteria-infected lungs, Figure S17; optimized ABD-AMP conjugate improves longitudinal on-target accumulation of active AMP in PAO1-infected thighs, Figure S18; ABD-AMP exhibits on-target activation across multiple dose range while retaining lung-to-off-target organ selectivity, Figure S19; (ABD)_2_-(EEG)_6_-S12-(d)KLA-Cy7 increases accumulation of active (d)KLA-Cy7 in the infected lungs while reducing exposure in kidneys and liver, Figure S20; (ABD)_2_-(EEG)_6_-S12-(d)Pex exhibits a good safety profile based on serum chemistry analysis and body weight change, Figure S21; (ABD)_2_-(EEG)_6_-S12-(d)Pex exhibits a good safety profile based on serum analysis of pro-inflammatory cytokines, Figure S22; (ABD)_2_-(EEG)_6_-S12-(d)Pex exhibits a good safety profile based on histology evaluation, Figure S23; (ABD)_2_-(EEG)_6_-S12-(d)KLA exhibits a good safety profile, Figure S24; LC-MS analysis of (d)Pex-azide, Figure S25; LC-MS analysis of ABD-(d)Pex conjugates, Table S1; selectivity indices (IC50/MIC and HE50/MIC) of ABD-AMP conjugates, Table S2; list of peptide substrates and sequences, Table S3; pharmacokinetics evaluation of ABD-AMP conjugates, Table S4; AUC table of longitudinal biodistribution study in PAO1-infected mice, Table S5; list of AMPs and sequences, Table S6; list of ABD-AMP conjugates, sequences, and molecular weights (PDF)

## ABBREVIATIONS

ABD: albumin-binding domain
AMP: antimicrobial peptide
