# Supplementary material for: Conditional Antimicrobial Peptide Therapeutics
Source: ACS Nano. 2022 Aug 18;16(10):15779–91. doi: 10.1021/acsnano.2c04162 (PMC9619929; doi:10.1021/acsnano.2c04162)
Supplement: Supplementary file 1 — nn2c04162_si_001.pdf [file nn2c04162_si_001.pdf]

Supporting Information

Conditional antimicrobial peptide therapeutics

Chayanon Ngambenjawong[1,2], *Leslie W. Chan*[1,3†], *Heather E. Fleming*[1,4], *Sangeeta N. Bhatia*[1,2,4-7]*

[1]Koch Institute for Integrative Cancer Research, Massachusetts Institute of Technology, Cambridge, MA 02139, USA.

[2]Institute for Medical Engineering and Science, Massachusetts Institute of Technology, Cambridge, MA 02139, USA.

[3†]Wallace H. Coulter Department of Biomedical Engineering, Georgia Institute of Technology and Emory School of Medicine, Atlanta, GA 30332, USA.

[4]Howard Hughes Medical Institute, Cambridge, MA 02139, USA.

[5]Department of Electrical Engineering and Computer Science, Massachusetts Institute of Technology, Cambridge, MA 02139, USA.

[6]Department of Medicine, Brigham and Women's Hospital and Harvard Medical School, Boston MA 02115, USA.

[7]Broad Institute of Massachusetts Institute of Technology and Harvard, Cambridge, MA 02139, USA.

†Present address

Supplementary methods

Evaluation of FRET substrate cleavage kinetics

FRET peptide substrates (15 µL, 20 µM in PBS) were incubated with an equal volume of thrombin solution (25 nM in PBS) (with or without human serum albumin (HSA) (500 µM)) or mouse serum at RT and monitored for Cy5 fluorescence for 4 h using a plate reader. After 4 h, a concentrated thrombin solution (1 µL at 0.47 mg/mL) was added to the reaction wells and incubated for additional 2 h to fully cleave the substrate to derive at the final fluorescence readout for normalization. HSA additive was confirmed to be absent of contaminant proteases by assessing with FRET S12 substrate.

Evaluation of conjugate cleavage kinetics

ABD-(D)Pex-Cy7 conjugates (40 µL, 20 µM in PBS) were incubated with an equal volume of thrombin solution (25 nM in PBS) (with or without HSA (500 µM)) or mouse serum at RT. At different time points, aliquots (5 µL) were drawn, mixed with 15 µL of PBS (supplemented with Halt™ protease inhibitor cocktail and EDTA), and stored in a -20 °C freezer. The samples were analyzed by SDS-PAGE to quantify % cleavage based on Cy7 signals of the intact conjugate and the released (D)Pex-Cy7.

Synthesis of MSA-Cy7-(D)Pex

A solution of mouse serum albumin (MSA) in PBS was incubated with Cy7-NHS ester (1 eq.) at RT for 2 h. Excess dye was removed via 50 kDa Amicon® centrifugal filter (10 times). MSA-Cy7 was then reacted with DBCO-sulfo NHS ester (2 eq.) at RT for 2 h. Excess crosslinker was removed via 50 kDa Amicon® centrifugal filter (10 times). Finally, MSA-Cy7-DBCO was reacted with (D)pexiganan-azide (2 eq.) at RT for 24 h. The excess peptide was removed via 50 kDa Amicon® centrifugal filter (10 times).

Bio-layer interferometry (BLI)

BLI was performed on an Octet® RED96 (ForteBio). ABD-$(EEG)_6$-S12-Biotin (100 nM) or $(ABD)_2$-$(EEG)_6$-S12-Biotin (75 nM) were immobilized to streptavidin tips for 5 min, followed by a 5-min baseline wash. Two-fold serial dilutions of human serum albumin (HSA) or MSA, starting from 62.5 and 125 nM respectively, were prepared and used for the association step which spanned 15 min and was followed by the dissociation step for 15 min. PBS (0.05 % Tween-20) was used for preparation of all protein solutions. Binding parameters were analyzed on Octet® analysis studio software.

Size exclusion chromatography

HSA, ABD-AMP conjugates, and their mixtures were analyzed on an NGC™ liquid chromatography system (Bio-Rad, CA, USA) using an ENrich™ SEC 650 10 x 300 Column (Bio-Rad, CA, USA) with PBS as a running buffer at 0.75 mL/min flow rate.

Mouse model of thigh infection

11-12-week-old CD-1 mice were rendered neutropenic by intraperitoneal injections of cyclophosphamide at 4 d (150 mg/kg) and 1 d (100 mg/kg) prior to infection. PAO1 inoculation was performed by intramuscular injection of PAO1 suspension (1 x 10^5 cfu in 50 µL PBS) into both thighs of the mice via 29G insulin needles.

Supplementary figures

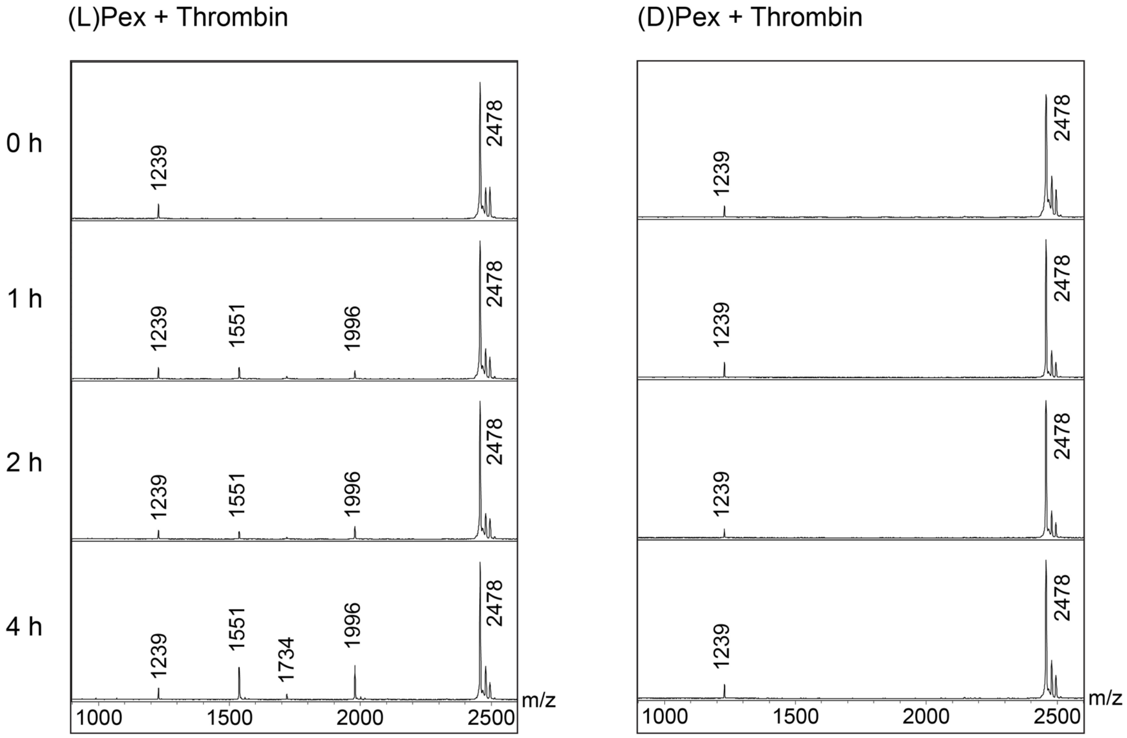

2478: (L)Pex: $[M + H]^+$: NH_2-GIGKFLKKAKKFGKAFVKILKK-$CONH_2$
(D)Pex: $[M + H]^+$: NH_2-GiGkflkkakkfGkafvkilkk-$CONH_2$

1239: (L)Pex: $[M + 2H]^{2+}$: NH_2-GIGKFLKKAKKFGKAFVKILKK-$CONH_2$
(D)Pex: $[M + 2H]^{2+}$: NH_2-GiGkflkkakkfGkafvkilkk-$CONH_2$

1996: Truncated (L)Pex: $[M + H]^+$: NH_2-GIGKFLKKAKKFGKAFVK~~ILKK-CONH2~~

1734: Truncated (L)Pex: $[M + H]^+$: ~~NH2-GIGKFLK~~KAKKFGKAFVKILKK-$CONH_2$

1551: Truncated (L)Pex: $[M + H]^+$: NH_2-GIGKFLKKAKKFGK~~AFVKILKK-CONH2~~

Figure S1. Stability of (L)Pex and (D)Pex in thrombin.

Stability of (L)Pex (Left) and (D)Pex (Right) in thrombin. (L)Pex was susceptible to cleavage by thrombin. Its degraded peptide fragments are denoted at the bottom. No cleavage of (D)Pex was observed.

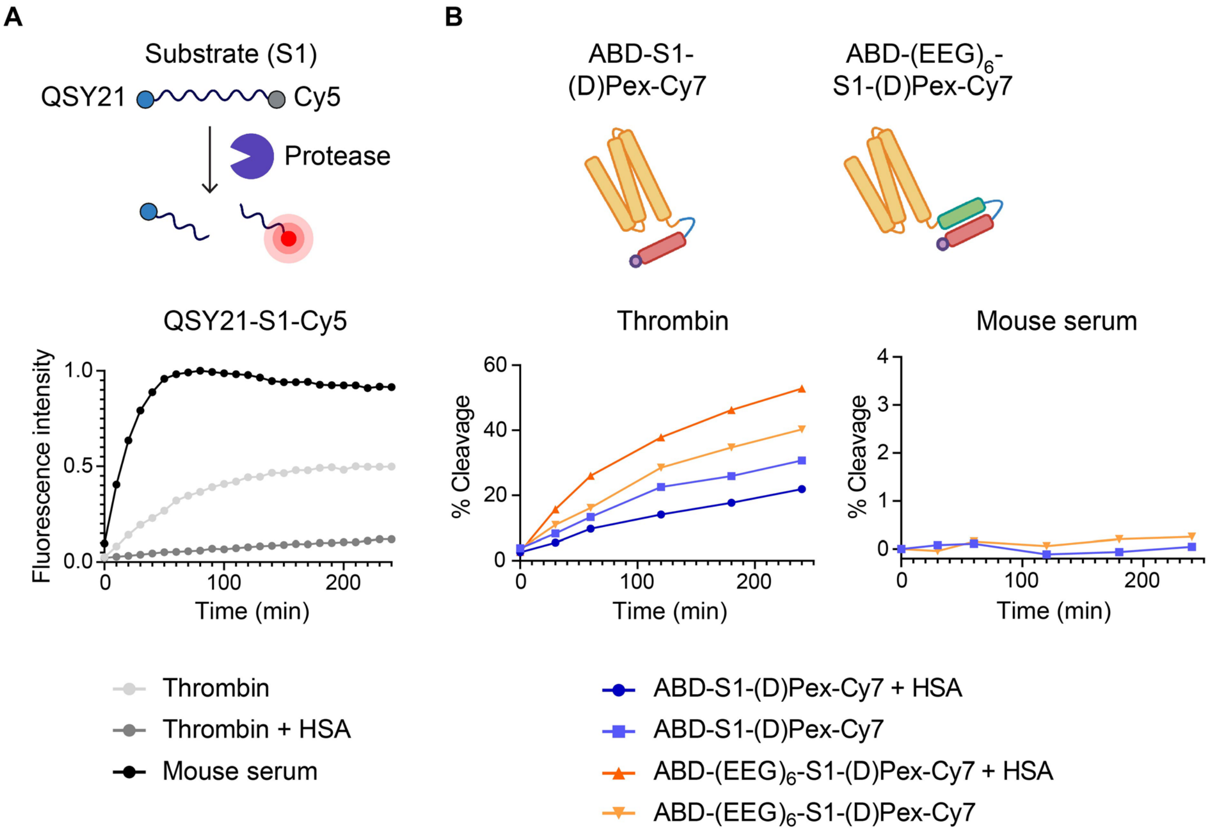

Figure S2. Albumin association and anionic block affect cleavage kinetics of ABD-S1-AMP conjugates by thrombin and mouse serum.

(A) Evaluation of cleavage kinetics of FRET peptide substrate S1 by thrombin (with or without HSA) and mouse serum. QSY21-S1-Cy5 was rapidly cleaved in mouse serum. The presence of HSA retarded cleavage kinetics of the FRET substrate by thrombin. (B) Evaluation of cleavage kinetics of ABD-AMP-Cy7 conjugates in thrombin (left) and mouse serum (right). Cleavage rate of ABD-S1-(D)Pex-Cy7 was slower in the presence of HSA. Cleavage rate of ABD-(EEG)$_6$-S12-(D)Pex-Cy7 was faster than that of ABD-S1-(D)Pex-Cy7 and was further increased in the presence of HSA. Both conjugates had high stability in mouse serum.

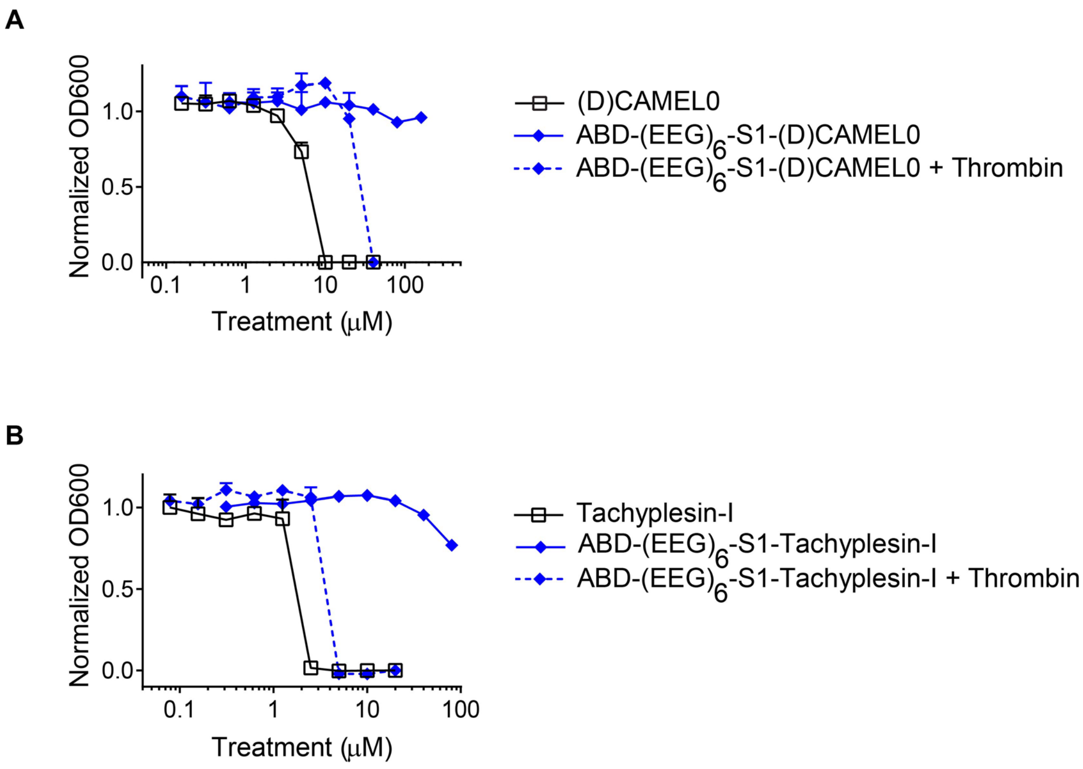

Figure S3. ABD-AMP conjugates exhibit activity masking across multiple model AMPs. Antibacterial activity masking of (A) ABD-$(EEG)_6$-S1-(D)CAMEL0 and (B) ABD-$(EEG)_6$-S1-Tachyplesin I was assessed via microdilution assays on PAO1. Bacteria viabilities following conjugate treatments were measured based on bacterial turbidity at 600 nm (OD600) and normalized to the non-treated control.

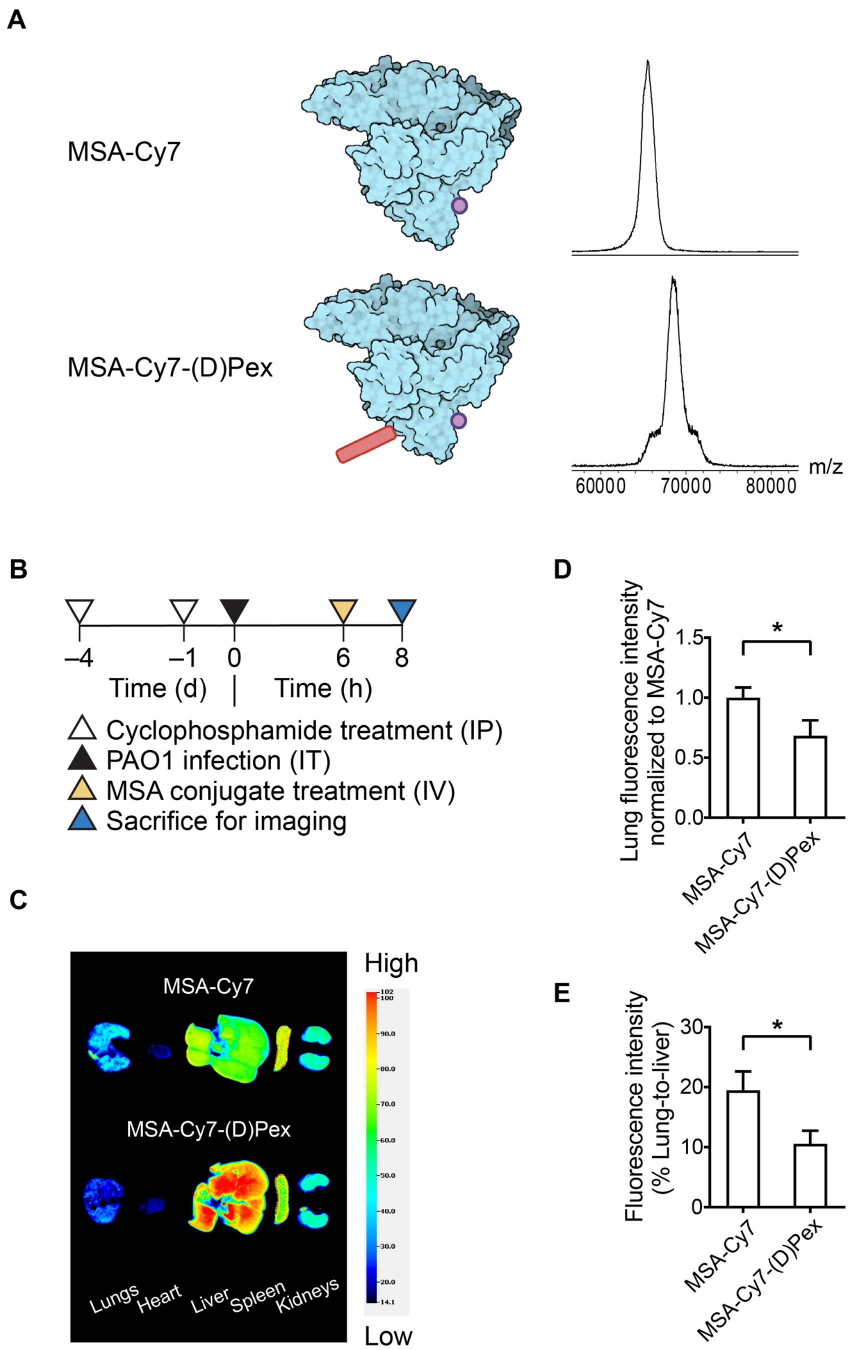

Figure S4. Conjugation of (D)Pex to MSA increased liver accumulation.
(A) Schematics (left) and mass spectra (right) of MSA-Cy7 and MSA-Cy7-(D)Pex. (B) Experimental timeline for biodistribution study. (C) Representative *ex vivo* fluorescence images of MSA accumulation in different organs imaged on an Odyssey CLx imager. (n = 3). (D) Quantification of total lung fluorescence signals normalized to MSA-Cy7 control. (E) Quantification of lung-to-liver fluorescence signals. (D and E) were plotted as mean ± SD (n = 3) and analyzed with unpaired t-test. * denotes statistical significance ($P < 0.05$). IP: Intraperitoneal injection, IT: Intratracheal instillation, IV: Intravenous injection.

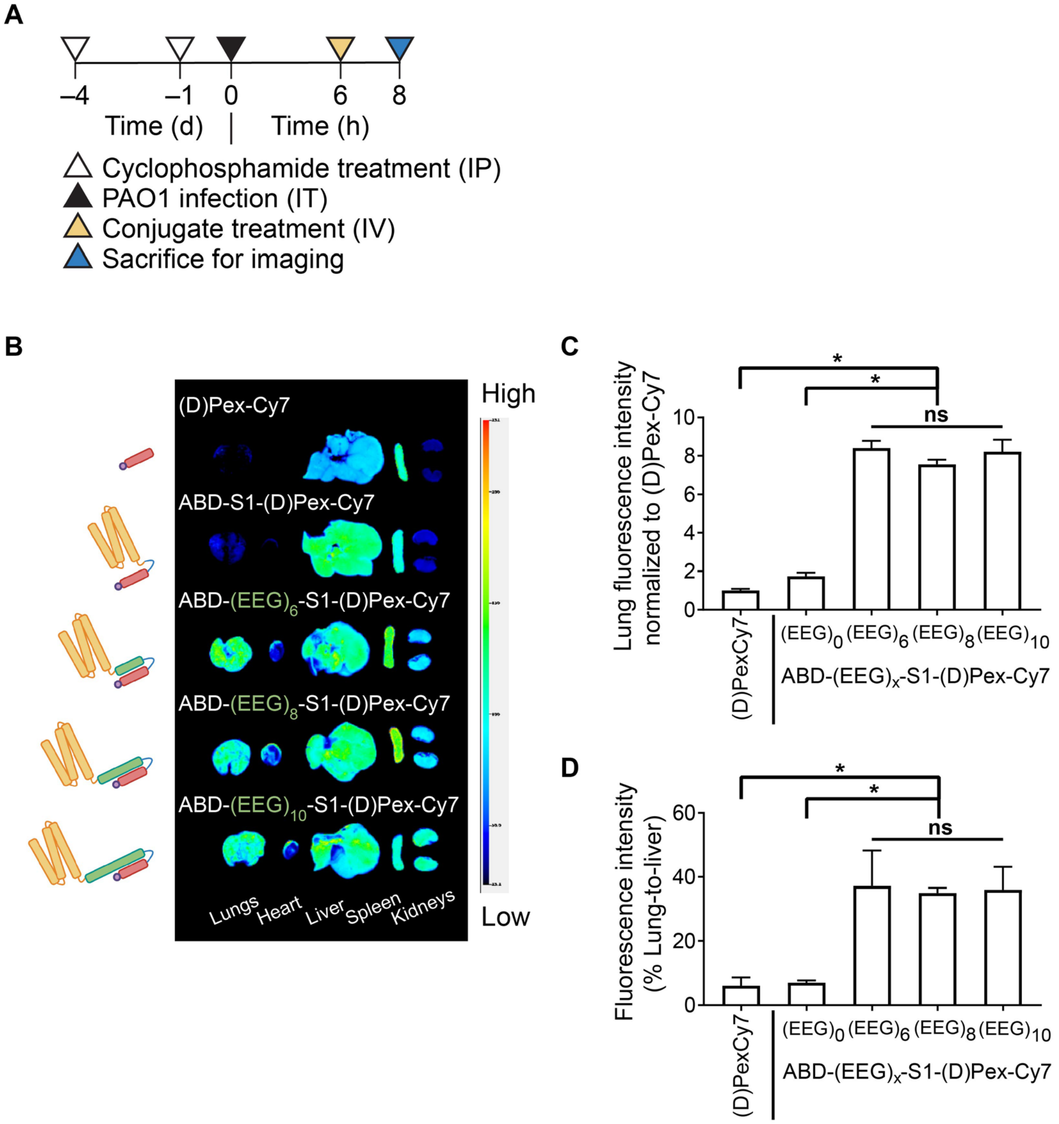

Figure S5. Improvement in biodistribution of ABD-AMP conjugate is saturated after reaching the threshold anionic block length.

(A) Experimental timeline for biodistribution study of ABD-$(EEG)_x$-S1-(D)Pex-Cy7 conjugates with varying anionic block lengths (B) Representative *ex vivo* fluorescence images of conjugate accumulation in different organs imaged on an Odyssey CLx imager. (n = 3). (C) Quantification of total lung fluorescence signals normalized to (D)Pex-Cy7 control group. (D) Quantification of lung-to-liver fluorescence signals. (C and D) were plotted as mean ± SD and analyzed with One-way ANOVA with Tukey post-hoc tests. * denotes statistical significance ($P < 0.05$). IP: Intraperitoneal injection, IT: Intratracheal instillation, IV: Intravenous injection.

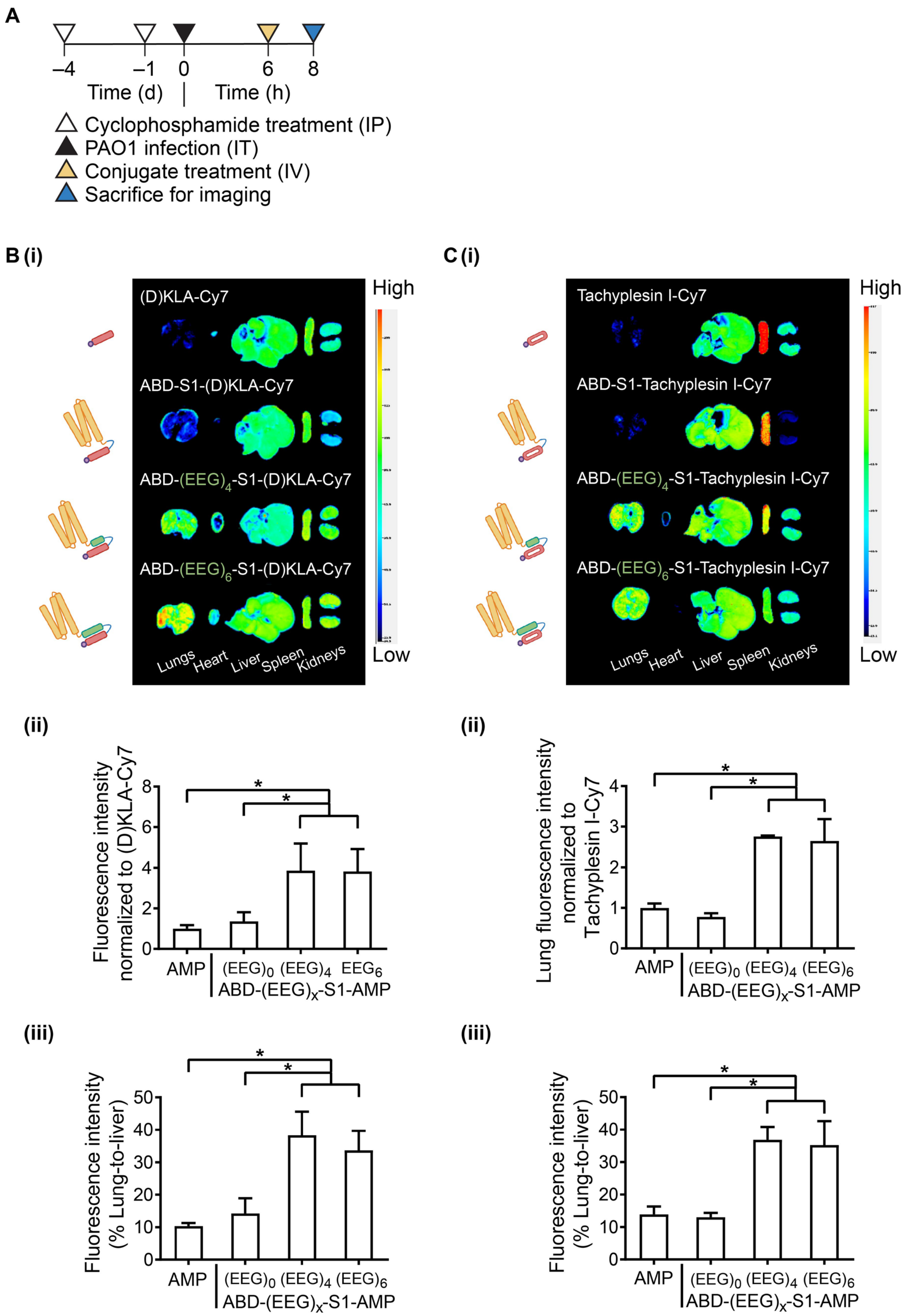

A
–4
–1
0
6
8
Time (d)
Time (h)
Cyclophosphamide treatment (IP)
PAO1 infection (IT)
Conjugate treatment (IV)
Sacrifice for imaging
B (i)
(D)KLA-Cy7
ABD-S1-(D)KLA-Cy7
ABD-(EEG)4-S1-(D)KLA-Cy7
ABD-(EEG)6-S1-(D)KLA-Cy7
Lungs Heart Liver Spleen Kidneys
High
Low
C (i)
Tachyplesin I-Cy7
ABD-S1-Tachyplesin I-Cy7
ABD-(EEG)4-S1-Tachyplesin I-Cy7
ABD-(EEG)6-S1-Tachyplesin I-Cy7
Lungs Heart Liver Spleen Kidneys
High
Low
(ii)
Fluorescence intensity normalized to (D)KLA-Cy7
AMP
(EEG)0 (EEG)4 EEG6
ABD-(EEG)x-S1-AMP
(ii)
Lung fluorescence intensity normalized to Tachyplesin I-Cy7
AMP
(EEG)0 (EEG)4 (EEG)6
ABD-(EEG)x-S1-AMP
(iii)
Fluorescence intensity (% Lung-to-liver)
AMP
(EEG)0 (EEG)4 (EEG)6
ABD-(EEG)x-S1-AMP
(iii)
Fluorescence intensity (% Lung-to-liver)
AMP
(EEG)0 (EEG)4 (EEG)6
ABD-(EEG)x-S1-AMP

Figure S6. ABD-AMP conjugates with anionic block improves biodistribution of multiple model AMPs.

(A) Experimental timeline for biodistribution study of (B) ABD-$(EEG)_x$-S1-(D)KLA-Cy7 conjugates and (C) ABD-$(EEG)_x$-S1-Tachyplesin I-Cy7 conjugates. (i) Representative *ex vivo* fluorescence images of conjugate accumulation in different organs imaged on an Odyssey CLx imager. (n = 3 - 4). (ii) Quantification of total lung fluorescence signals normalized to the free AMP control group. (iii) Quantification of lung-to-liver fluorescence signals. (ii and iii) were plotted as mean ± SD and analyzed with One-way ANOVA with Tukey post-hoc tests. * denotes statistical significance ($P < 0.05$).

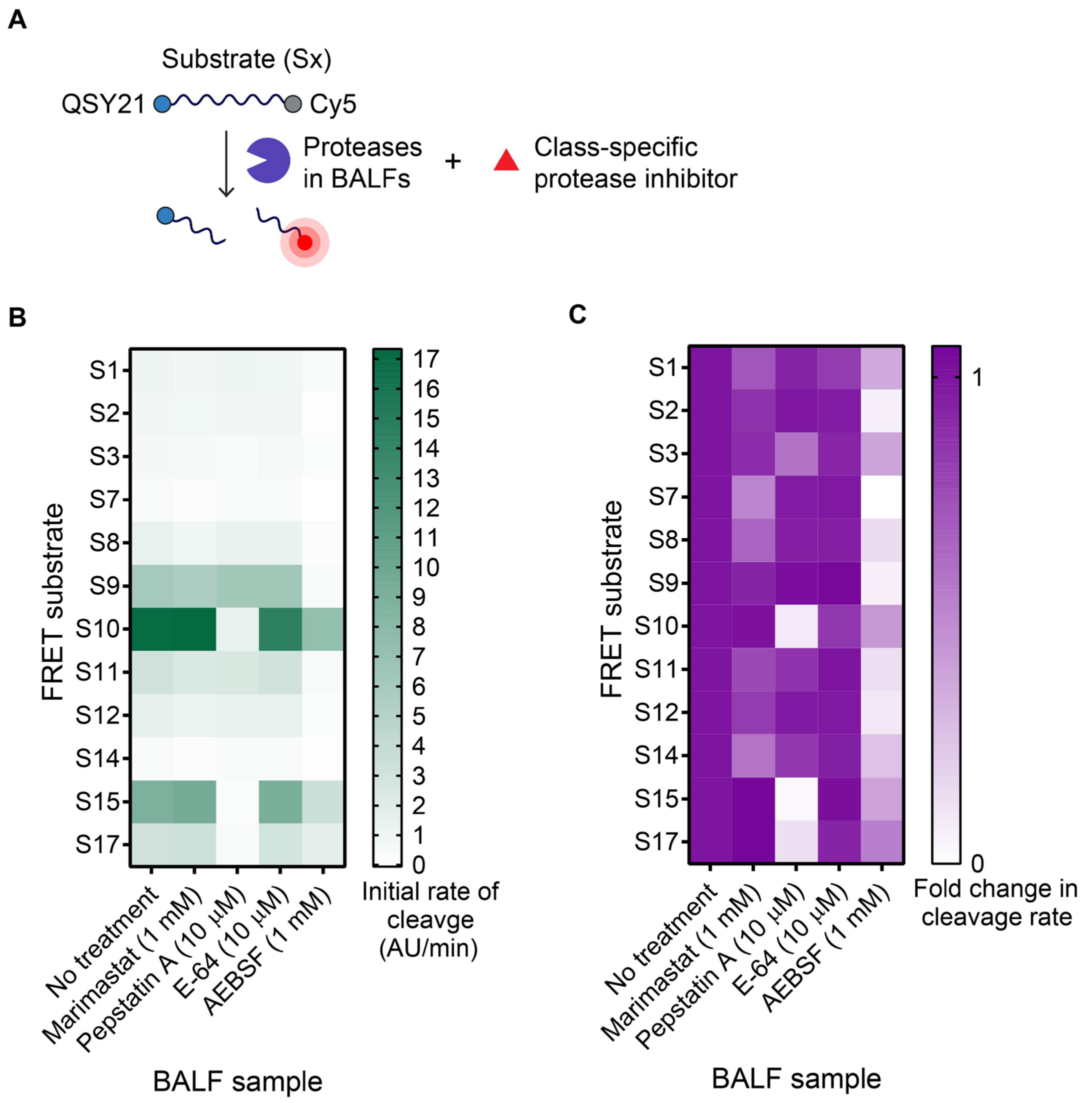

Figure S7. Proteolytic activities of BALFs on FRET substrates were inhibited by multiple class-specific protease inhibitors.

(A) Schematic of FRET substrate screening in PAO1-infected BALFs with protease inhibitors. FRET substrates were incubated in BALFs from PA-infected mice (10 h infection time point) supplemented with different protease inhibitors and monitored for cleavage via Cy5 fluorescence. (B) Heat map represents the average initial cleavage rates of each FRET substrate. (n = 2). (C) Normalized heat map relative to the no-inhibitor control.

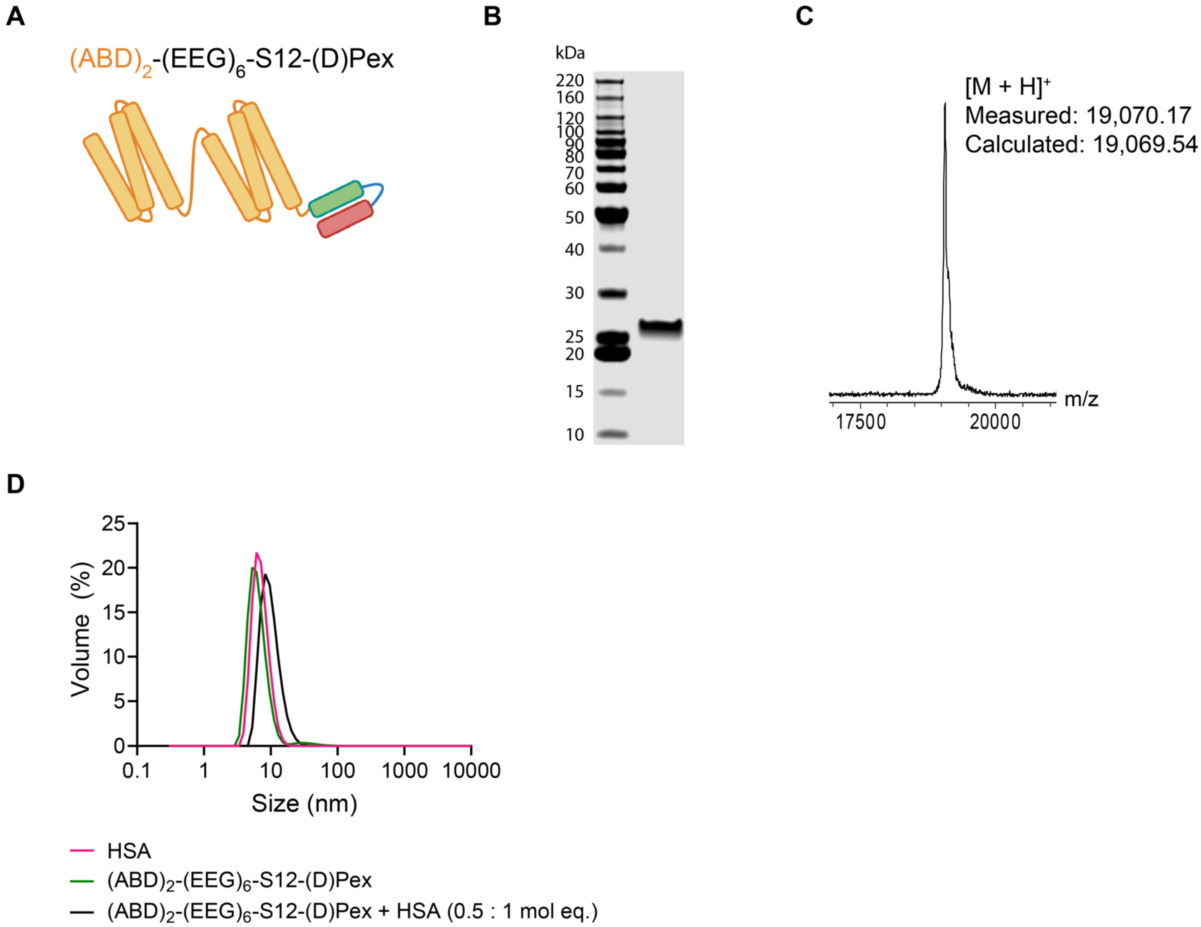

Figure S8. Schematic and characterization of divalent ABD-AMP conjugate.
(A) Schematic of $(ABD)_2$-$(EEG)_6$-S12-(D)Pex. (B) SDS-PAGE analysis of the conjugate detected with Coomassie blue staining and imaged on an Odyssey CLx imager (C) Molecular weight of the conjugate was measured via MALDI-ToF MS shown as mass-to-charge ratio (m/z). N-terminal methionine was spontaneously removed during ABD expression. (D) Dynamic light scattering (DLS) measurement of $(ABD)_2$-$(EEG)_6$-S12-(D)Pex, HSA, and their complex.

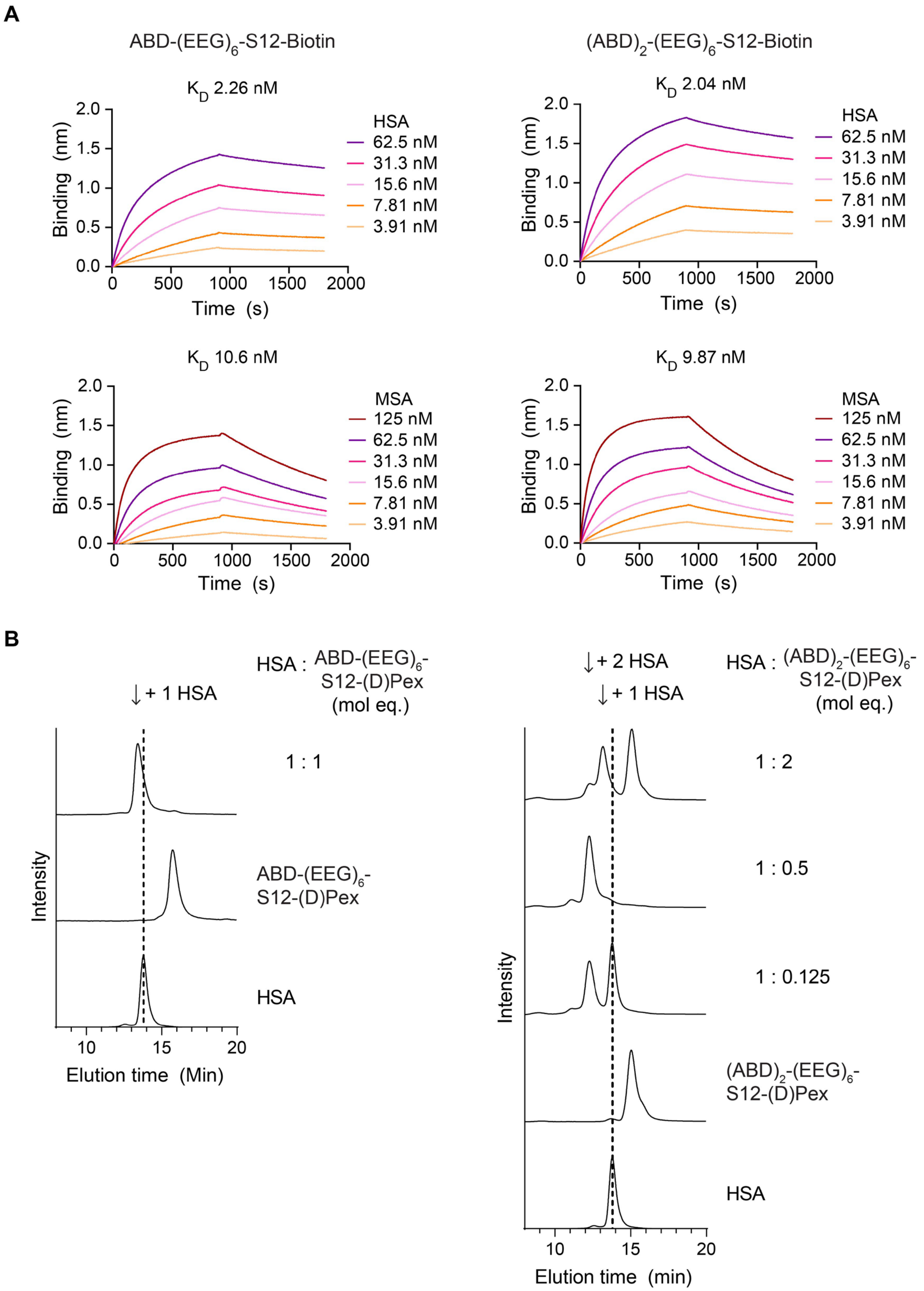
A
ABD-(EEG)6-S12-Biotin
(ABD)2-(EEG)6-S12-Biotin
KD 2.26 nM
KD 2.04 nM
KD 10.6 nM
KD 9.87 nM
Binding (nm)
Time (s)
HSA
MSA
125 nM
62.5 nM
31.3 nM
15.6 nM
7.81 nM
3.91 nM
B
HSA : ABD-(EEG)6-S12-(D)Pex (mol eq.)
HSA : (ABD)2-(EEG)6-S12-(D)Pex (mol eq.)
↓+ 1 HSA
↓+ 2 HSA
1 : 1
1 : 2
1 : 0.5
1 : 0.125
ABD-(EEG)6-S12-(D)Pex
(ABD)2-(EEG)6-S12-(D)Pex
HSA
Intensity
Elution time (Min)
Elution time (min)

Figure S9. Characterization of albumin binding by ABD and ABD-AMP conjugates.
(A) BLI measurement of apparent dissociation constants (K_D) of ABD-$(EEG)_6$-S12-Biotin (left) and $(ABD)_2$-$(EEG)_6$-S12-Biotin (right) on HSA (top) and MSA (bottom). (B) Size exclusion chromatography analysis of HSA association to ABD-$(EEG)_6$-S12-(D)Pex (left) and $(ABD)_2$-$(EEG)_6$-S12-(D)Pex (right).

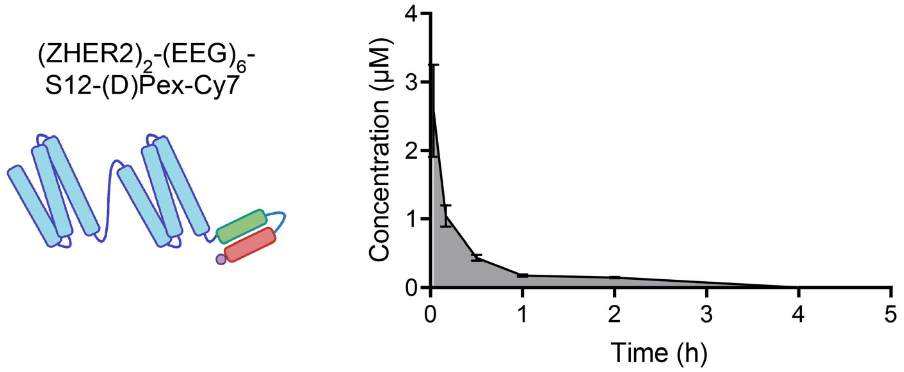

Figure S10. (ZHER2)$_2$-(EEG)$_6$-S12-(D)Pex-Cy7, as a non-albumin-binding control conjugate with anionic block, exhibits short circulation time.
Pharmacokinetics of (ZHER2)$_2$-(EEG)$_6$-S12-(D)Pex-Cy7. ZHER2 affibody was selected as a non-albumin-binding control with a similar three-helical protein domain as ABD.[1]

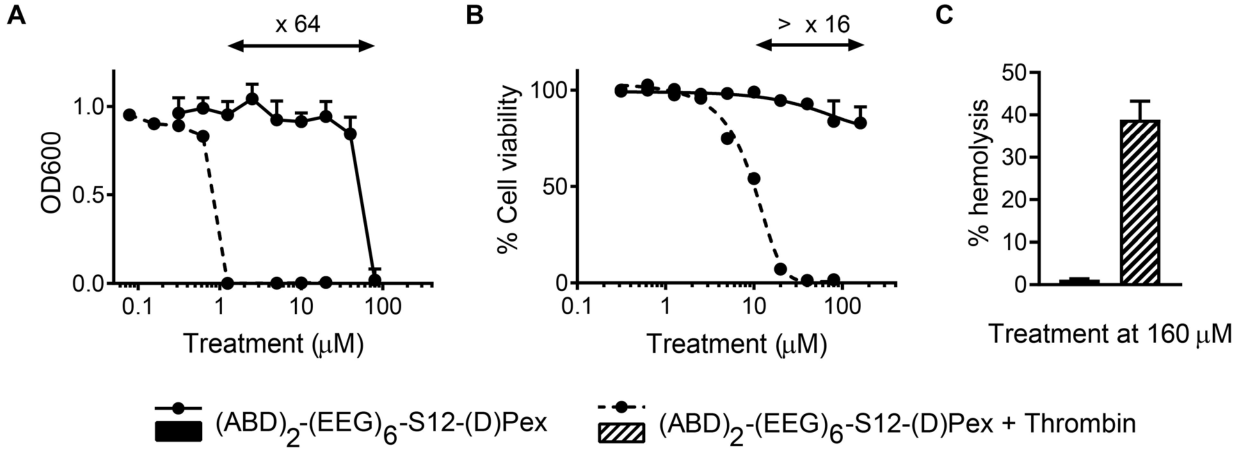

Figure S11. Divalent ABD-AMP conjugate exhibits enhanced activity masking.
(A) Antibacterial activity masking was assessed via microdilution assays on PAO1. Fold change in activity masking was based on the ratio of the minimum inhibitory concentrations (MIC) of the intact and cleaved conjugates. (B) Mammalian toxicity masking was assessed via MTS cell viability assay on L929 fibroblasts. Fold change in activity masking was based on the ratio of the 50 % inhibitory concentrations (IC50) of the intact and cleaved conjugates. (C) Hemolysis masking was assessed via hemolysis assay on mouse red blood cells.

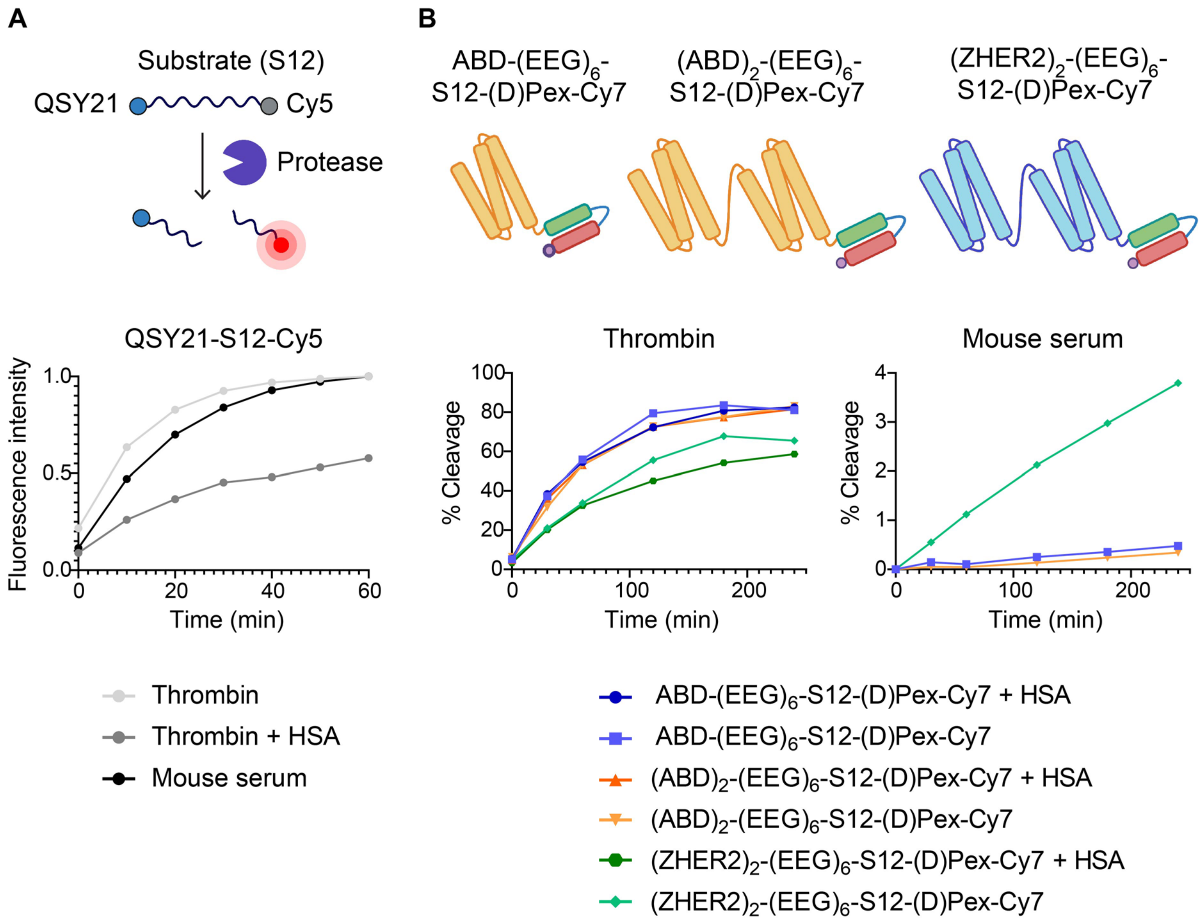

Figure S12. Divalent association with two albumins did not affect cleavage kinetics of (ABD)$_2$-(EEG)$_6$-S12-(D)Pex-Cy7.

(A) Evaluation of cleavage kinetics of FRET peptide substrate S12 by thrombin (with or without HSA) and mouse serum. QSY21-S12-Cy5 was rapidly cleaved in mouse serum. The presence of HSA retarded cleavage kinetics of the FRET substrate by thrombin. (B) Evaluation of cleavage kinetics of ABD-AMP-Cy7 and non-albumin-binding control (ZHER2) conjugates in thrombin (left) and mouse serum (right). Cleavage rates of ABD-(EEG)$_6$-S12-(D)Pex-Cy7 and (ABD)$_2$-(EEG)$_6$-S12-(D)Pex-Cy7 were comparable and were not affected by the presence of HSA. Both conjugates had high stability in mouse serum. (ZHER2)$_2$-(EEG)$_6$-S12-(D)Pex-Cy7 had a lower thrombin cleavage rate but was more susceptible to cleavage by mouse serum compared to the ABD-AMP conjugates.

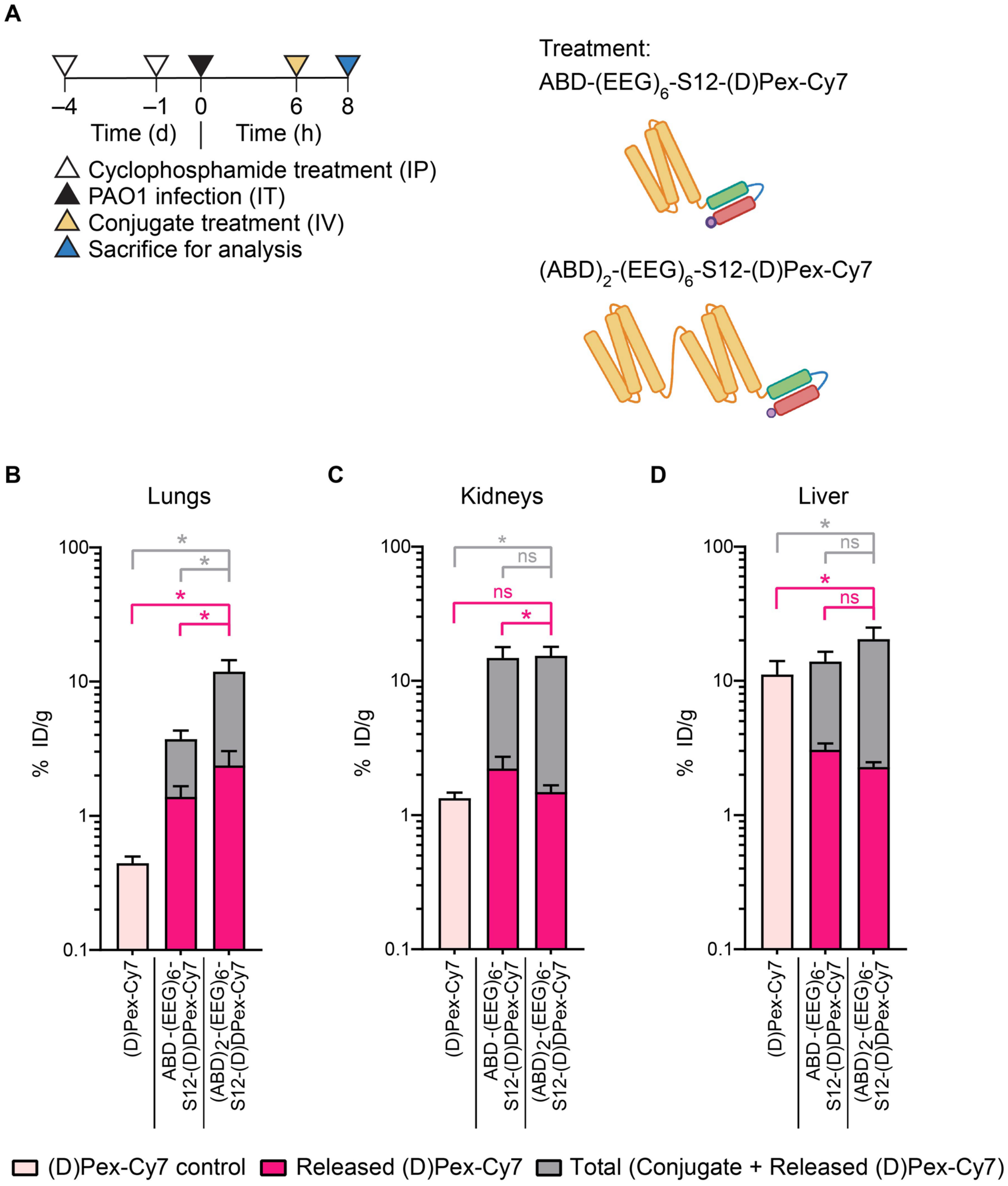

Figure S13. Divalent ABD-AMP conjugate delivers more active AMP to bacteria-infected lungs.

(A) Experimental timeline for biodistribution study comparing ABD-$(EEG)_6$-S12-(D)Pex-Cy7 and $(ABD)_2$-$(EEG)_6$-S12-(D)Pex-Cy7. Quantification of total and active fractions of the conjugates in (B) lungs, (C) kidneys, and (D) liver. (n = 3). (B - D) were plotted as mean ± SD and analyzed with One-way ANOVA with Tukey post-hoc tests. * denotes statistical significance ($P < 0.05$).

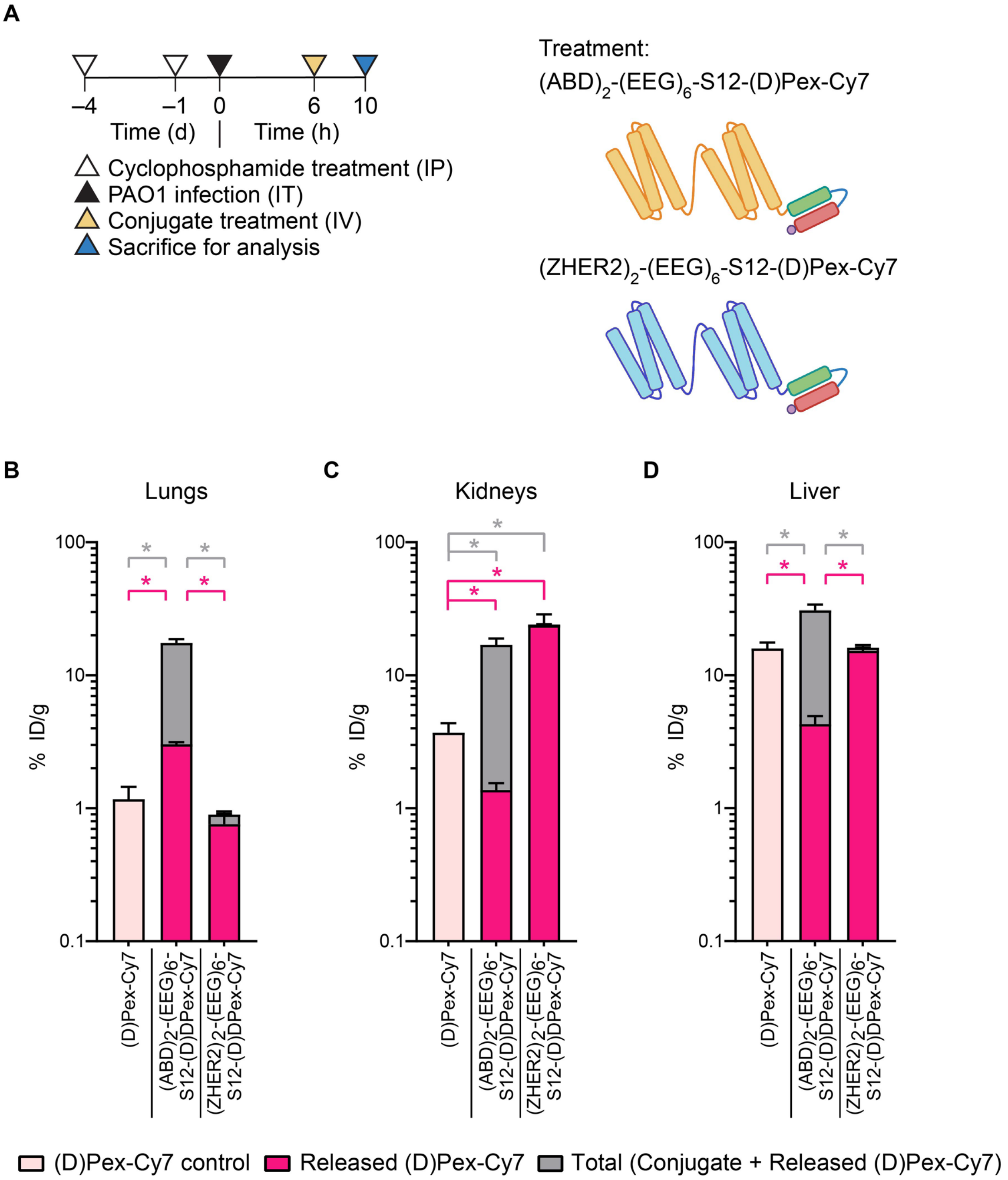

Figure S14. Albumin association of ABD-AMP conjugate is necessary for enhanced delivery of active AMP to bacteria-infected lungs.

(A) Experimental timeline for biodistribution study comparing $(ABD)_2$-$(EEG)_6$-S12-(D)Pex-Cy7 and non-albumin-binding $(ZHER2)_2$-$(EEG)_6$-S12-(D)Pex-Cy7. Quantification of total and active fractions of the conjugates in (B) lungs, (C) kidneys, and (D) liver. (n = 3). (B - D) were plotted as mean ± SD and analyzed with One-way ANOVA with Tukey post-hoc tests. * denotes statistical significance ($P < 0.05$).

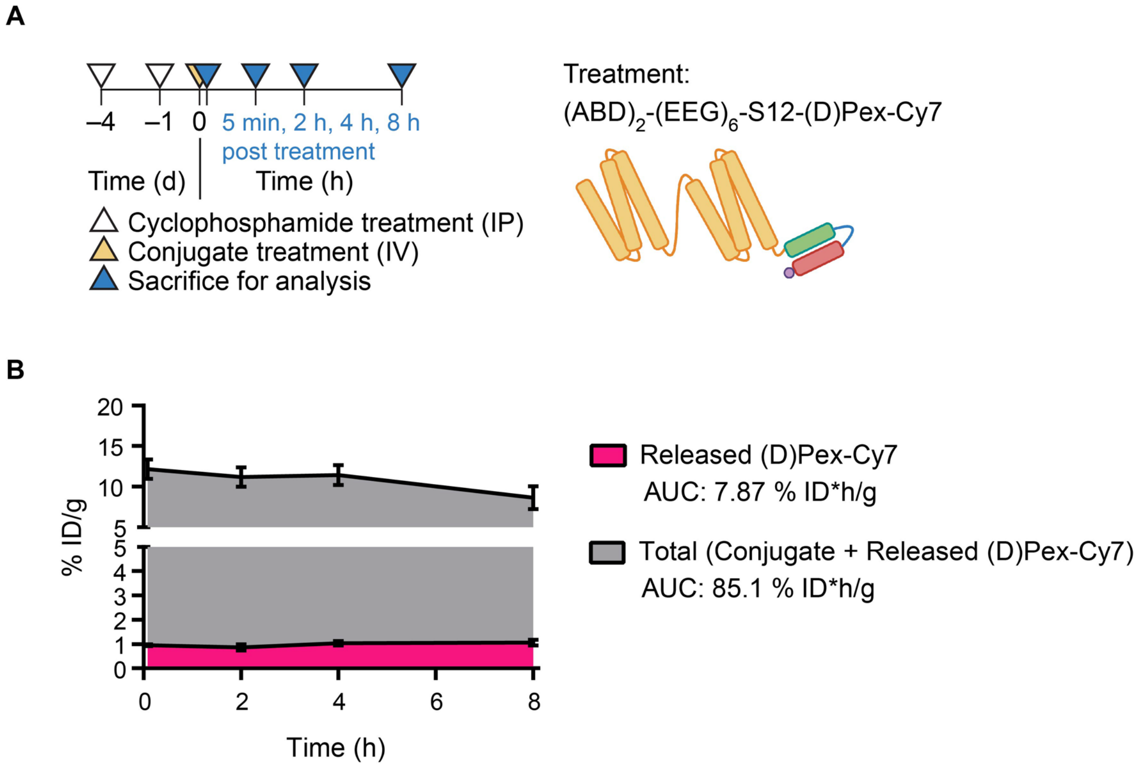

Figure S15. The level of active AMP remains steady in the non-infected lungs.
(A) Experimental timeline for longitudinal biodistribution study of $(ABD)_2$-S12-$(EEG)_6$-(D)Pex-Cy7 in non-infected, neutropenic mice (B) Accumulation over time of $(ABD)_2$-S12-$(EEG)_6$-(D)Pex-Cy7 (released and total fractions) in the lungs. (n = 3 - 4). (B) was plotted as mean % ID/g ± SD.

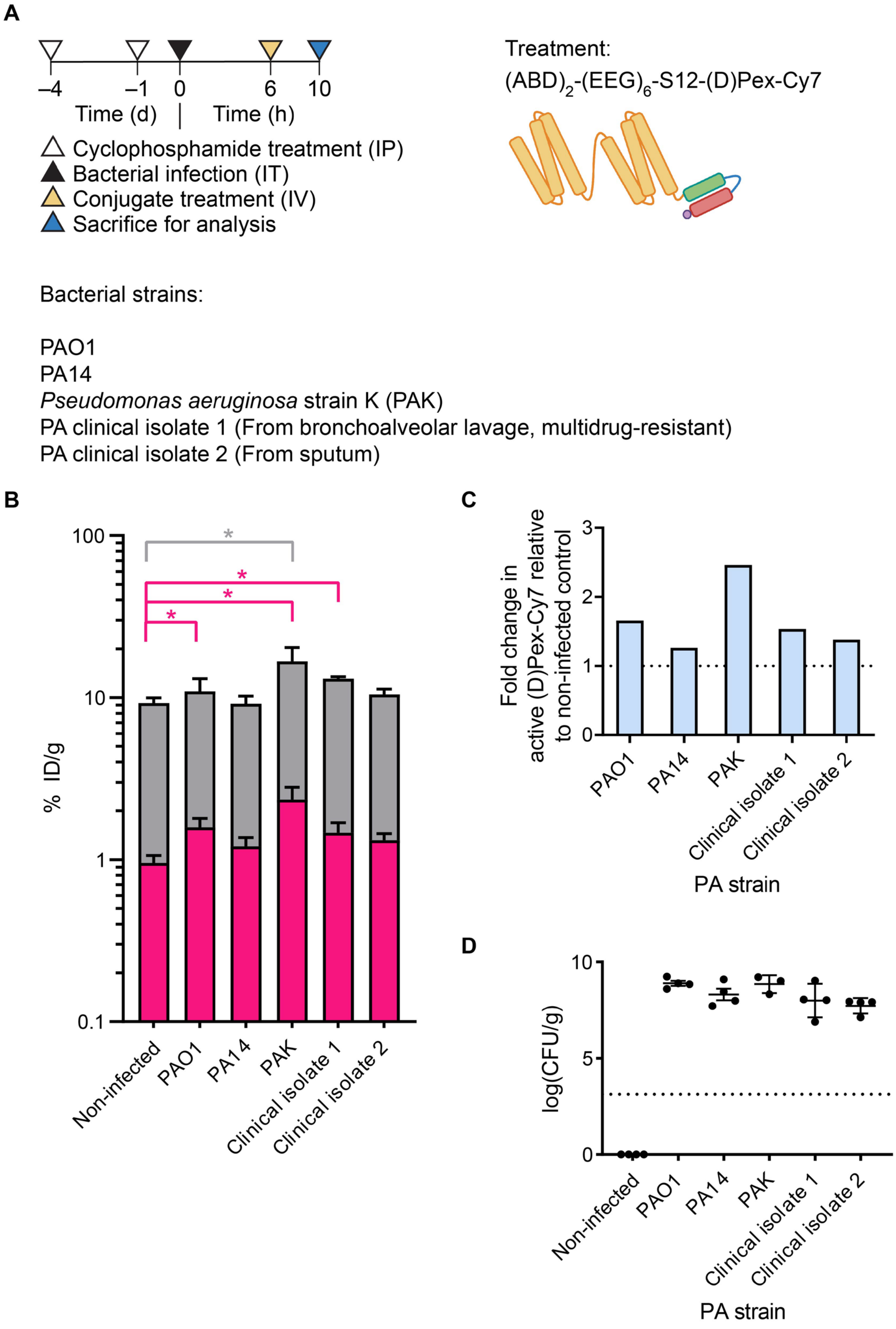
A
–4
–1
0
6
10
Time (d)
Time (h)
Cyclophosphamide treatment (IP)
Bacterial infection (IT)
Conjugate treatment (IV)
Sacrifice for analysis
Treatment:
(ABD)2-(EEG)6-S12-(D)Pex-Cy7
Bacterial strains:
PAO1
PA14
Pseudomonas aeruginosa strain K (PAK)
PA clinical isolate 1 (From bronchoalveolar lavage, multidrug-resistant)
PA clinical isolate 2 (From sputum)
B
% ID/g
100
10
1
0.1
Non-infected
PAO1
PA14
PAK
Clinical isolate 1
Clinical isolate 2
C
Fold change in active (D)Pex-Cy7 relative to non-infected control
3
2
1
0
PAO1
PA14
PAK
Clinical isolate 1
Clinical isolate 2
PA strain
D
log(CFU/g)
10
5
0
Non-infected
PAO1
PA14
PAK
Clinical isolate 1
Clinical isolate 2
PA strain

Figure S16. ABD-AMP conjugate is preferentially activated in bacteria-infected lungs. (A) Experimental timeline for biodistribution study evaluating activation of $(ABD)_2$-$(EEG)_6$-S12-(D)Pex-Cy7 in different lung infection models. Mice were infected with 2 x 10^5 cfu of bacteria for all strains. (B) Quantification of total and active fractions of the conjugate in lungs (n = 3 - 4). (B) was plotted as mean ± SD and analyzed with One-way ANOVA with Tukey post-hoc tests. * denotes statistical significance ($P < 0.05$). (C) Fold change in released (D)Pex-Cy7 in the infected lungs relative to the non-infected control. (D) Bacteria burden in the lungs at the study endpoint presented as mean of log(CFU/g) ± SD. Dotted line indicates limit of detection.

A

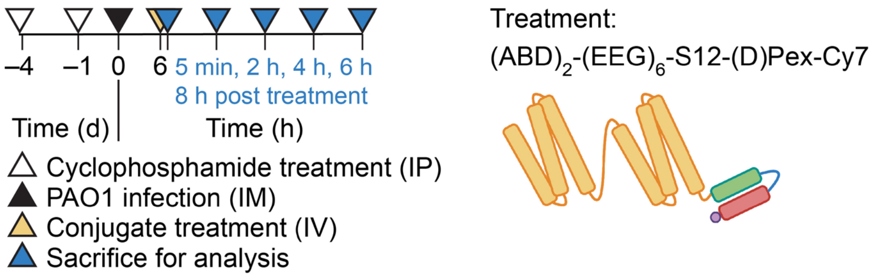

B

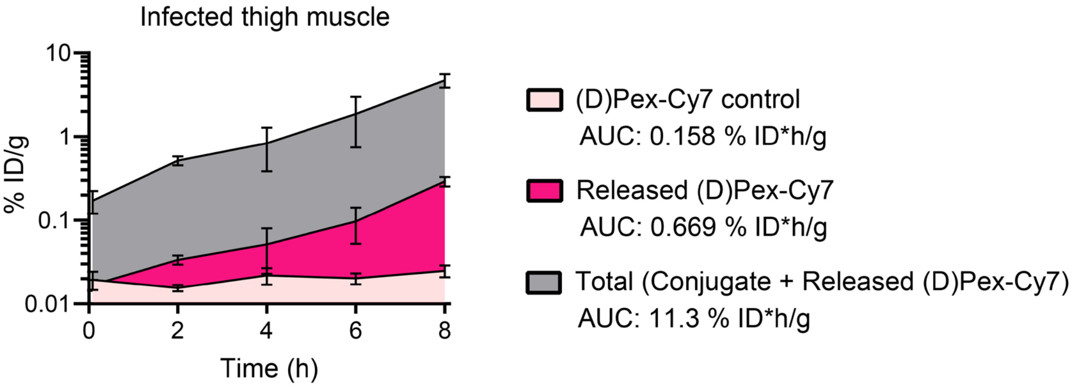

Figure S17. Optimized ABD-AMP conjugate improves longitudinal on-target accumulation of active AMP in PAO1-infected thighs.
(A) Experimental timeline for longitudinal biodistribution study of $(ABD)_2$-S12-$(EEG)_6$-(D)Pex-Cy7 in a PAO1 thigh infection model (B) Accumulation over time of $(ABD)_2$-S12-$(EEG)_6$-(D)Pex-Cy7 (released and total fractions) in the infected thigh muscle. (n = 4). (B) was plotted as mean % ID/g ± SD. IM: Intramuscular injection.

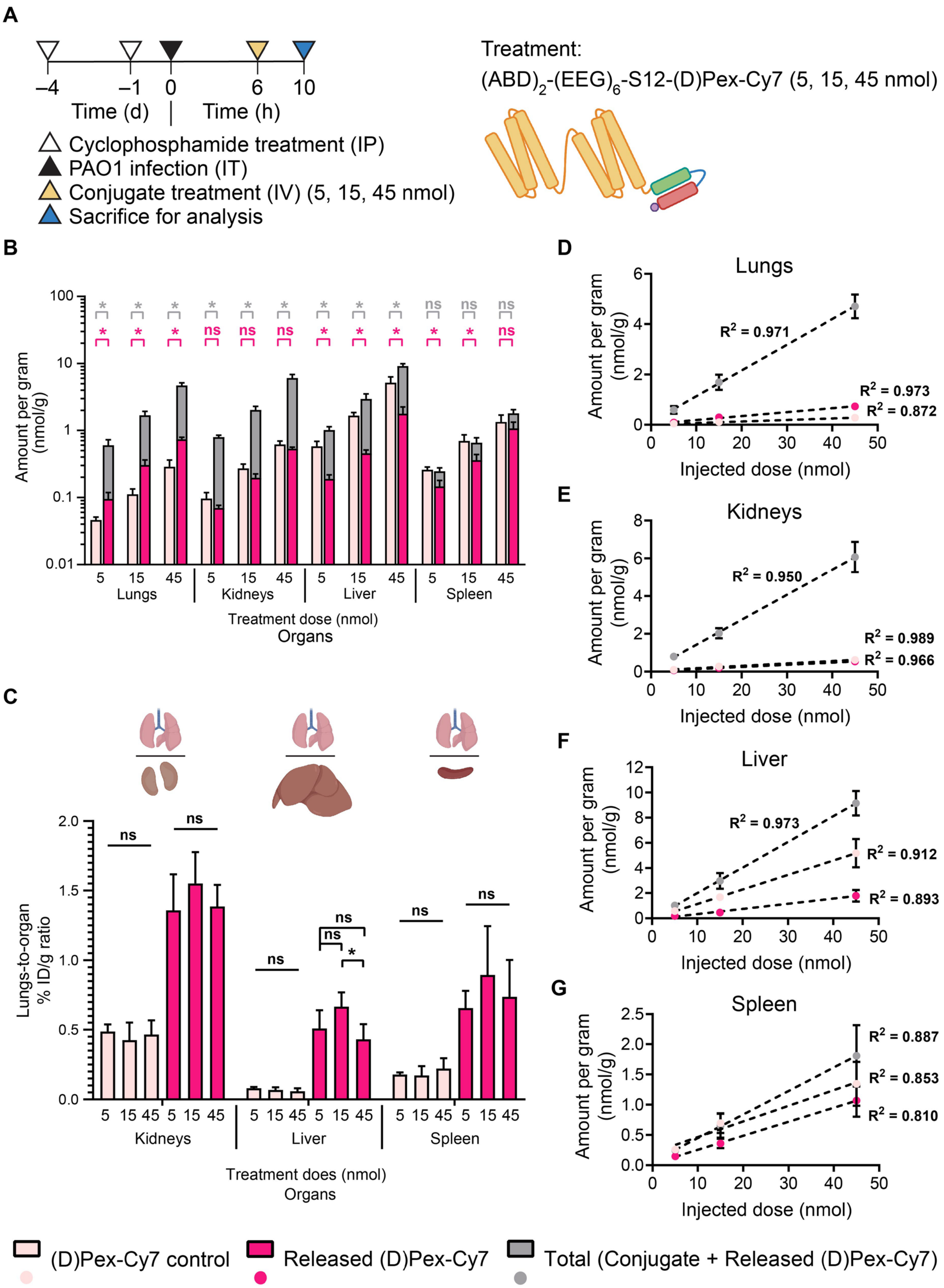
A
−4
−1
0
6
10
Time (d)
Time (h)
Cyclophosphamide treatment (IP)
PAO1 infection (IT)
Conjugate treatment (IV) (5, 15, 45 nmol)
Sacrifice for analysis
Treatment:
(ABD)2-(EEG)6-S12-(D)Pex-Cy7 (5, 15, 45 nmol)
B
Amount per gram (nmol/g)
Treatment dose (nmol)
Organs
Lungs
Kidneys
Liver
Spleen
ns
*
C
Lungs-to-organ % ID/g ratio
Treatment does (nmol)
Organs
D
Lungs
E
Kidneys
F
Liver
G
Spleen
Injected dose (nmol)
R2 = 0.971
R2 = 0.973
R2 = 0.872
R2 = 0.950
R2 = 0.989
R2 = 0.966
R2 = 0.973
R2 = 0.912
R2 = 0.893
R2 = 0.887
R2 = 0.853
R2 = 0.810
(D)Pex-Cy7 control
Released (D)Pex-Cy7
Total (Conjugate + Released (D)Pex-Cy7)

Figure S18. ABD-AMP exhibits on-target activation across multiple dose range while retaining lung-to-off-target organ selectivity.

(A) Experimental timeline for biodistribution study of $(ABD)_2$-S12-$(EEG)_6$-(D)Pex-Cy7 at 5, 15, and 45 nmol. (B) Quantification of total and active fractions of $(ABD)_2$-S12-$(EEG)_6$-(D)Pex-Cy7 and (D)Pex-Cy7 (C) Lung-to-organ % ID/g ratio plot. Linear regression between the injected dose and organ accumulation of $(ABD)_2$-S12-$(EEG)_6$-(D)Pex-Cy7 and (D)Pex-Cy7 in (D) lungs, (E) kidneys, (F) liver, and (G) spleen. (n = 4) (B and C) were plotted as mean ± SD and analyzed with One-way ANOVA with Tukey post-hoc tests. * denotes statistical significance ($P < 0.05$).

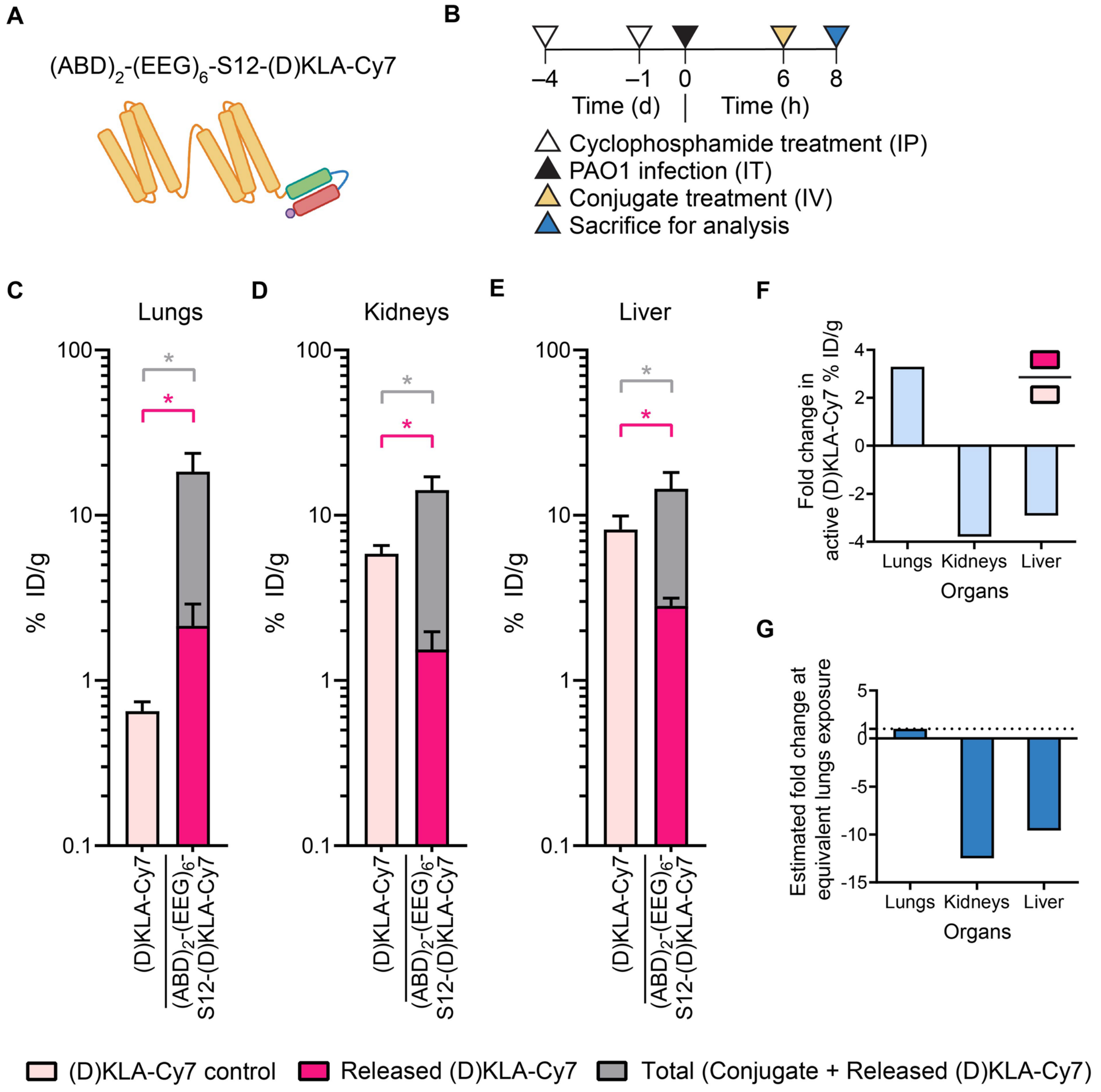

Figure S19. (ABD)$_2$-(EEG)$_6$-S12-(D)KLA-Cy7 increases accumulation of active (D)KLA-Cy7 in the infected lungs while reducing exposure in kidneys and liver.

(A) Schematics of divalent (ABD)$_2$-(EEG)$_6$-S12-(D)KLA-Cy7. (B) Experimental timeline for biodistribution study. Quantification of total and active fractions of (ABD)$_2$-S12-(EEG)$_6$-(D)KLA-Cy7 and (D)KLA-Cy7 in (C) lungs, (D) kidneys, and (E) liver. (n = 4). (C - E) were plotted as mean ± SD and analyzed with One-way ANOVA with Tukey post-hoc tests. * denotes statistical significance ($P < 0.05$). (F) Fold change in % ID/g of the released (D)KLA-Cy7 fraction of the conjugate relative to the free (D)KLA-Cy7 control in different organs. Reduced accumulations relative to the control were plotted as negative fold change. (G) Estimated fold change in % ID/g of the released (D)KLA-Cy7 fraction of the conjugate relative to the free (D)KLA-Cy7 control in off-target organs at equivalent lung exposure.

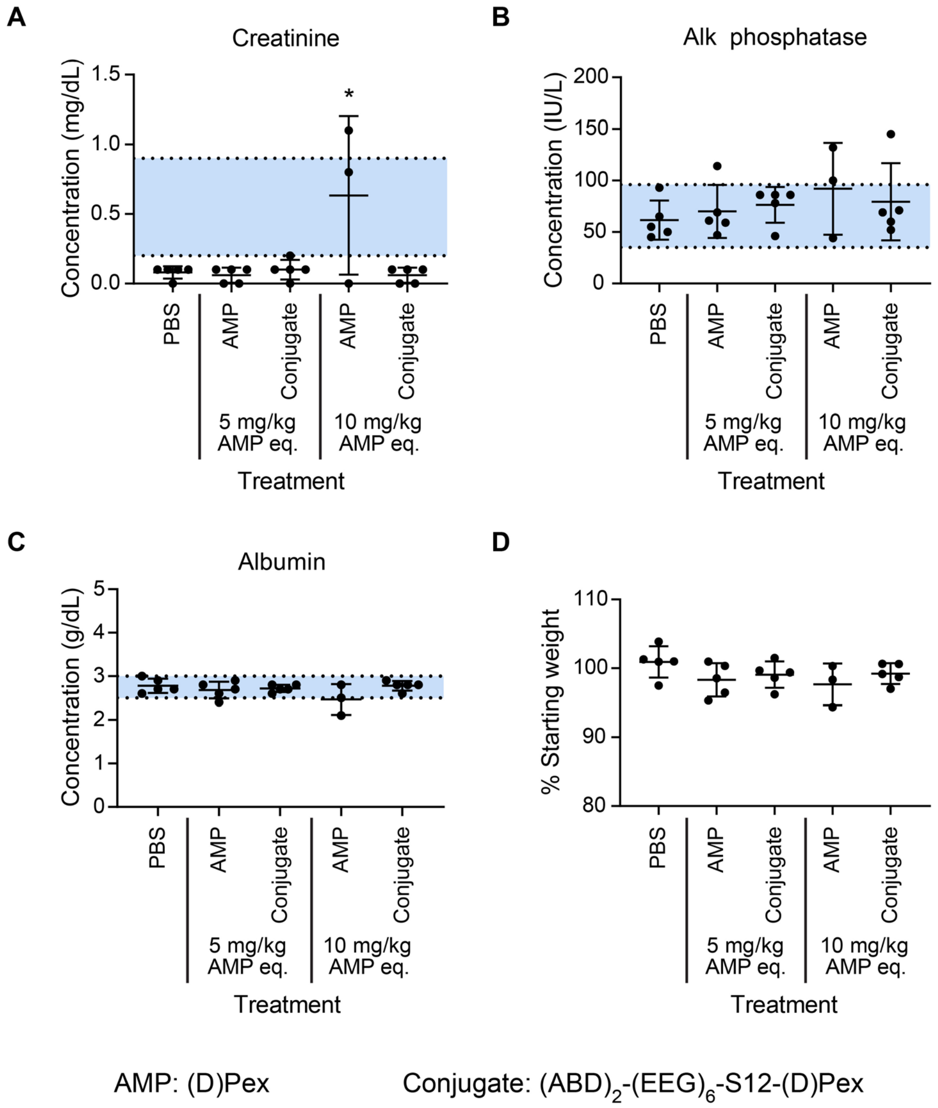

Figure S20. $(ABD)_2$-$(EEG)_6$-S12-(D)Pex exhibits a good safety profile based on serum chemistry analysis and body weight change.

Serum chemistry analysis of the mice in different treatment groups. The serum levels are shown for (A) creatinine, (B) alkaline phosphatase, and (C) albumin. (D) Percent body weight at the study endpoint relative to the starting weight. (A - D) were plotted as mean ± SD and analyzed with One-way ANOVA with Tukey post-hoc tests. * denotes statistical significance ($P < 0.05$). (n = 3 - 5). Blue area indicates a normal reference range.

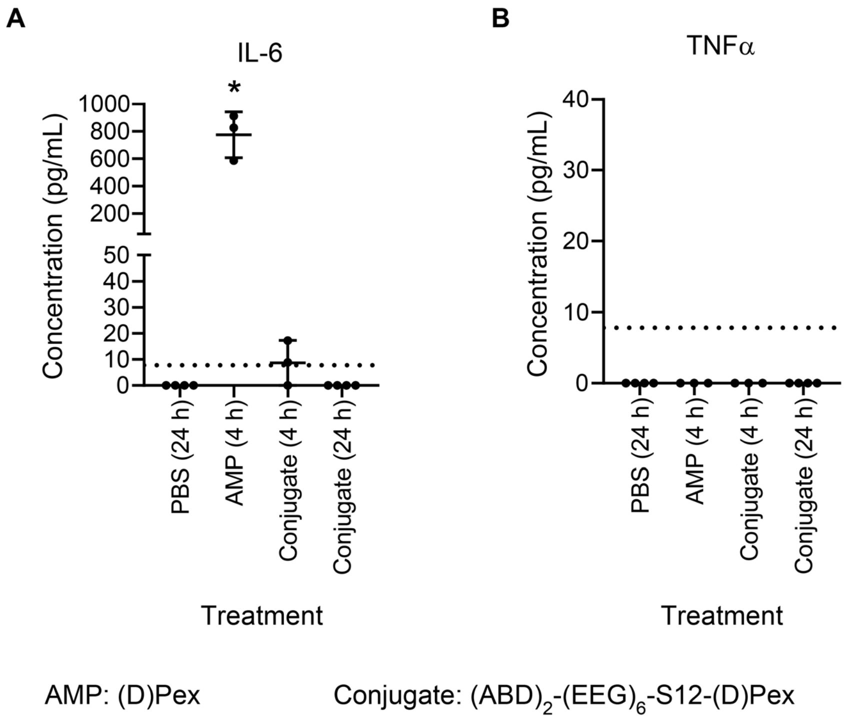

Figure S21. $(ABD)_2$-$(EEG)_6$-S12-(D)Pex exhibits a good safety profile based on serum analysis of pro-inflammatory cytokines.

ELISA-based quantification of serum pro-inflammatory cytokine (A) IL-6 and (B) TNF-α. Mice were intravenously administered (D)Pex or $(ABD)_2$-$(EEG)_6$-S12-(D)Pex at 10 mg/kg AMP eq. and euthanized for serum collection at 4 or 24 h. (A and B) were plotted as mean ± SD and analyzed with One-way ANOVA with Tukey post-hoc tests. * denotes statistical significance ($P < 0.05$). (n = 3 - 4). Dotted line indicates a limit of detection.

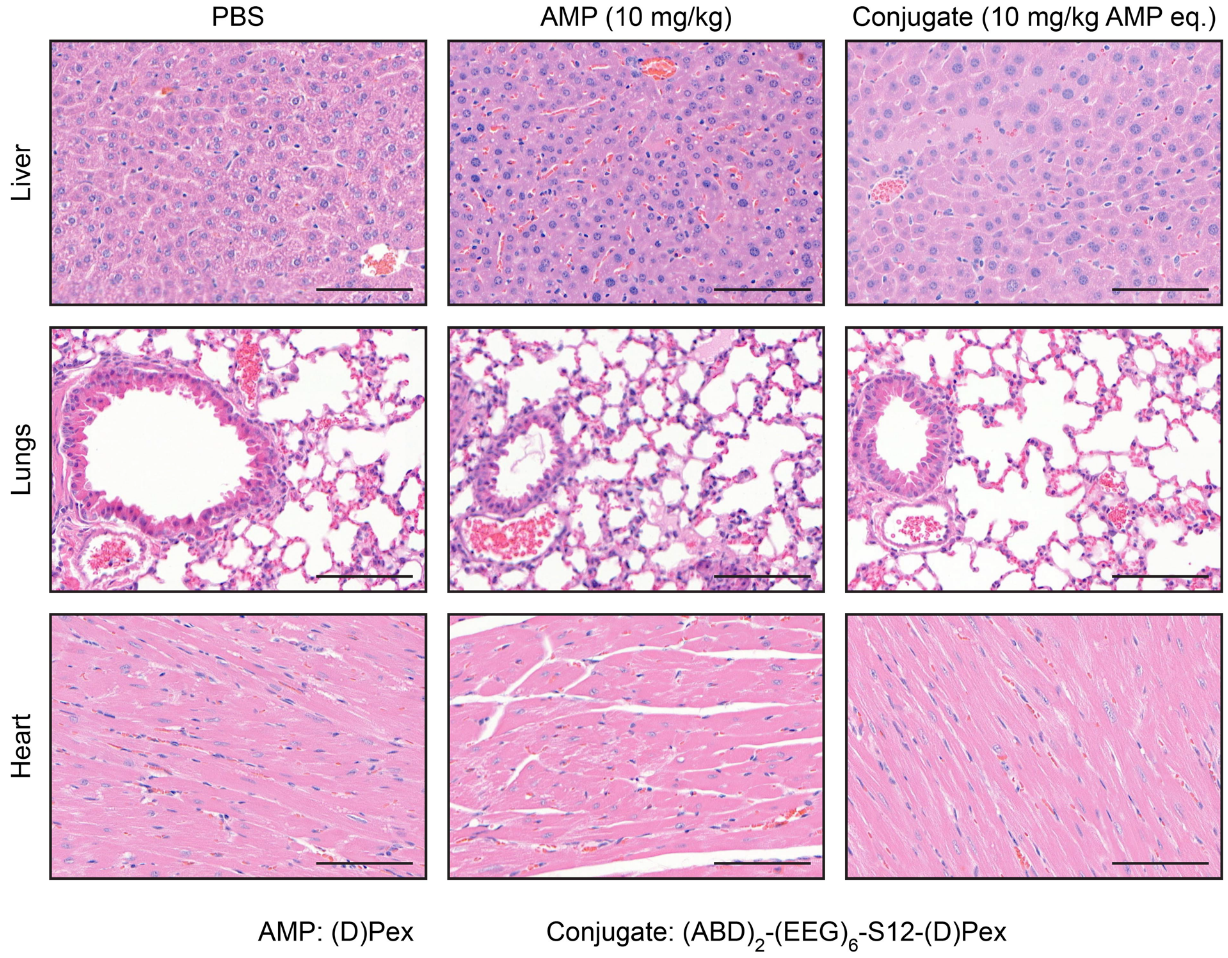

Figure S22. (ABD)$_2$-(EEG)$_6$-S12-(D)Pex exhibits a good safety profile based on histology evaluation.

H&E-stained sections of liver, lungs, and heart from the mice treated with PBS, (D)Pex, or (ABD)$_2$-(EEG)$_6$-S12-(D)Pex. (n = 3). Scale bar represents 100 μm.

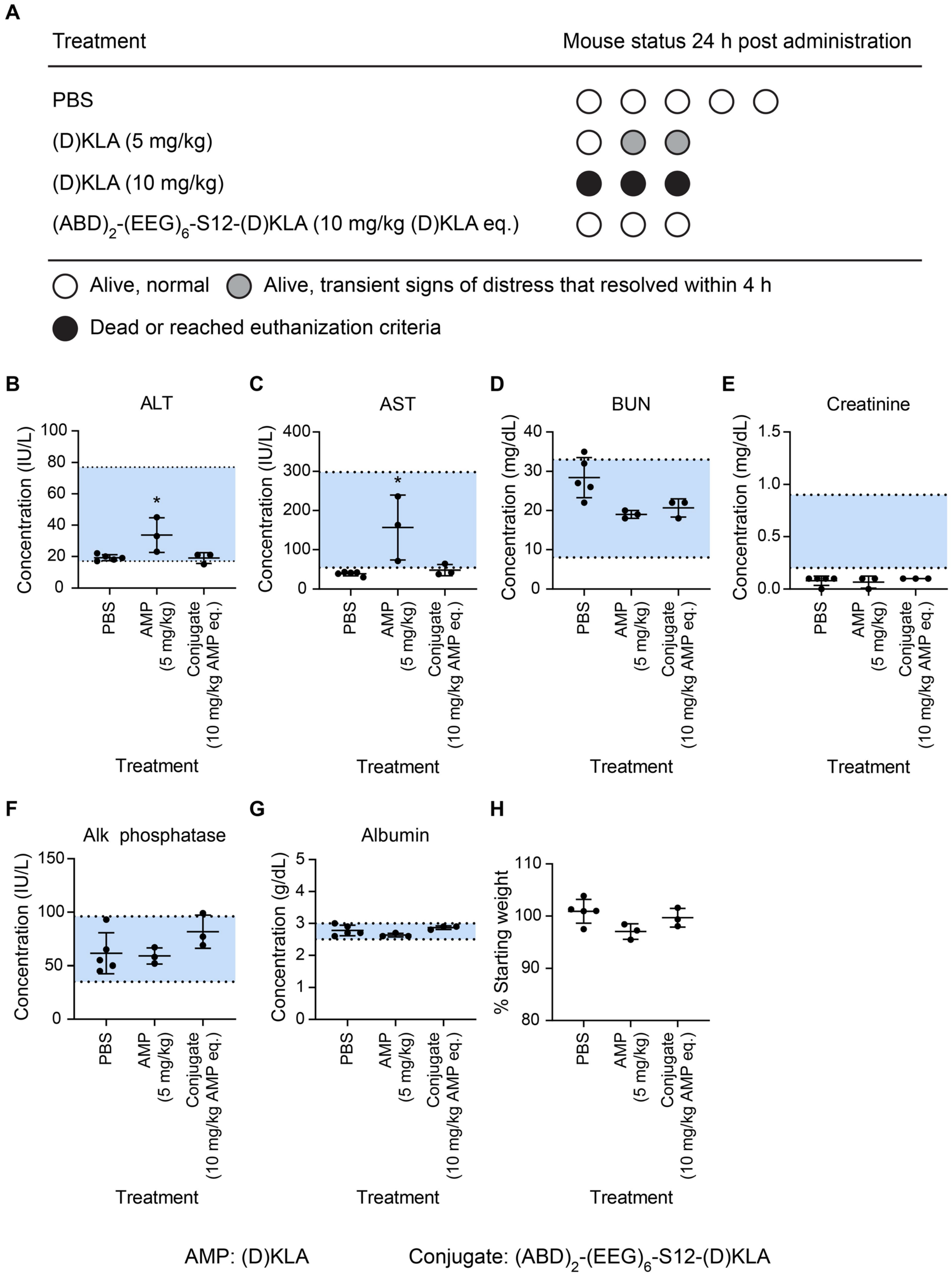
A
Treatment
Mouse status 24 h post administration
PBS
(D)KLA (5 mg/kg)
(D)KLA (10 mg/kg)
(ABD)$_2$-(EEG)$_6$-S12-(D)KLA (10 mg/kg (D)KLA eq.)
Alive, normal
Alive, transient signs of distress that resolved within 4 h
Dead or reached euthanization criteria
B
ALT
Concentration (IU/L)
C
AST
Concentration (IU/L)
D
BUN
Concentration (mg/dL)
E
Creatinine
Concentration (mg/dL)
F
Alk phosphatase
Concentration (IU/L)
G
Albumin
Concentration (g/dL)
H
% Starting weight
PBS
AMP (5 mg/kg)
Conjugate (10 mg/kg AMP eq.)
Treatment
AMP: (D)KLA
Conjugate: (ABD)$_2$-(EEG)$_6$-S12-(D)KLA

Figure S23. $(ABD)_2$-$(EEG)_6$-S12-(D)KLA exhibits a good safety profile.
(A) Treatment groups and status of the treated mice. Serum chemistry analysis of the mice in different treatment groups. Each circle represents a mouse. The serum levels are shown for (B) ALT, (C) AST, (D) BUN, (E) creatinine, (F) alkaline phosphatase, and (G) albumin. (H) Percent body weight at the study endpoint relative to the starting weight. (B - H) were plotted as mean ± SD and analyzed with One-way ANOVA with Tukey post-hoc tests. * denotes statistical significance ($P < 0.05$). (n = 3 - 5). Blue area indicates a normal reference range.

(D)Pex-azide

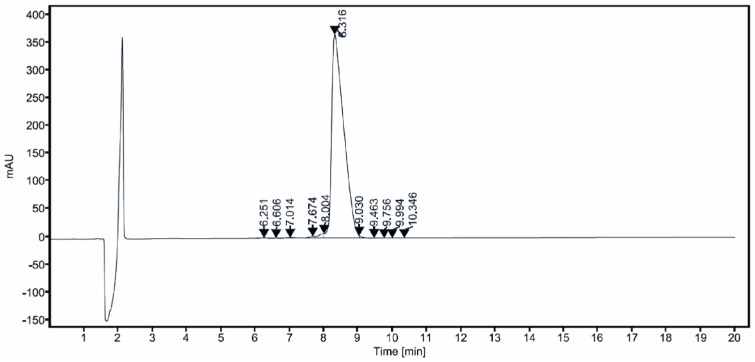

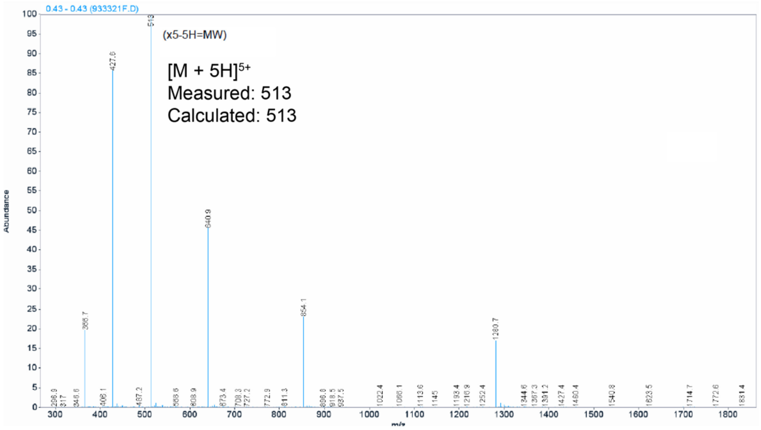

Figure S24. LC-MS analysis of (D)Pex-azide.

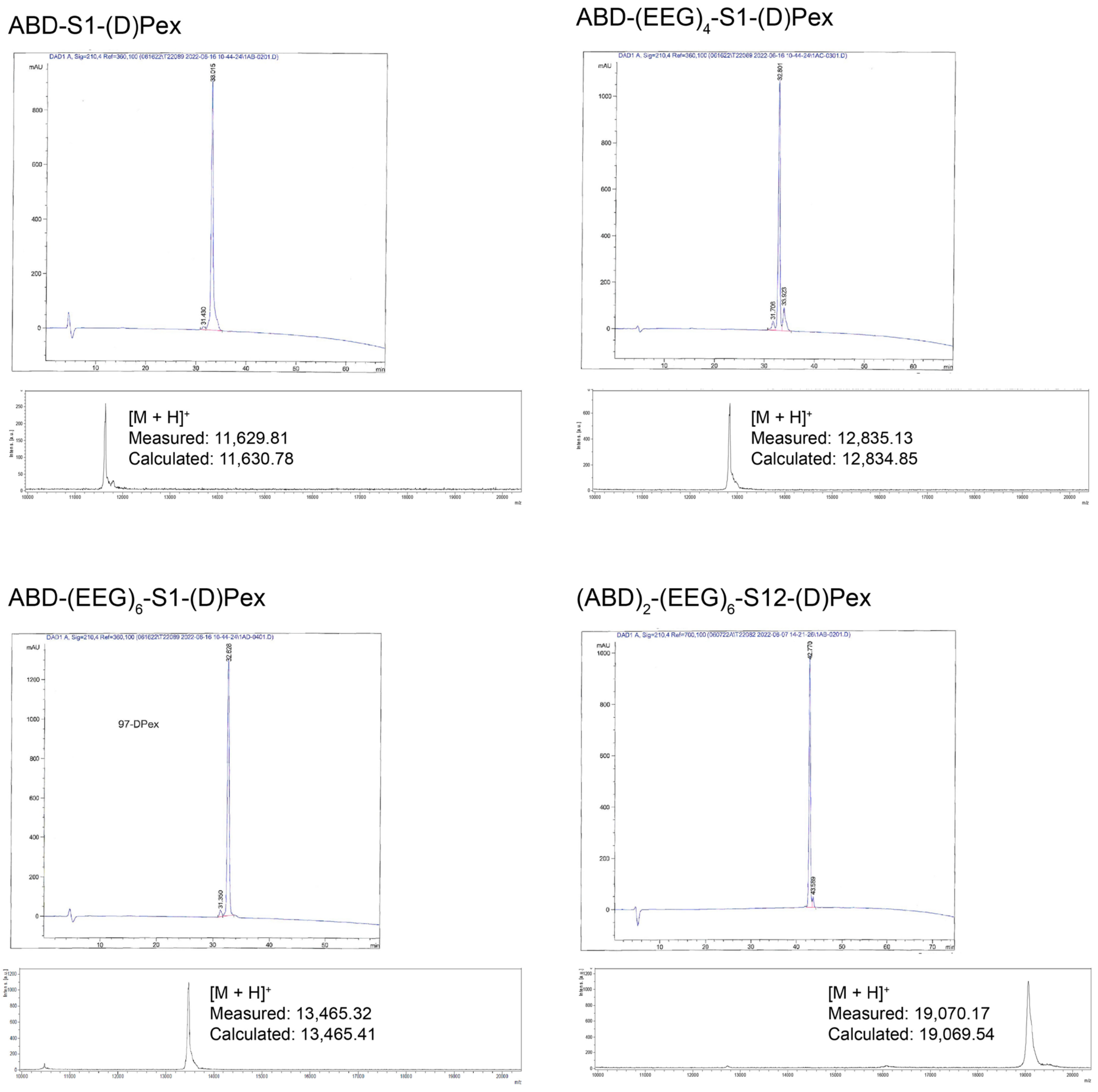

Figure S25. LC-MS analysis of ABD-(D)Pex conjugates.

Supplementary tables

Conjugate	MIC of cleaved conjugate on PAO1 (µM)	IC50 of intact conjugate on L929 (µM)	HE50* of intact conjugate (µM)	IC50/MIC	HE50/MIC
(D)Pex	1.25	7.13	> 160	5.7	> 128
ABD-S1-(D)Pex	1.25	64.91	> 160	51.9	> 128
ABD-$(EEG)_4$-S1-(D)Pex	1.25	77.89	> 160	62.3	> 128
ABD-$(EEG)_6$-S1-(D)Pex	1.25	80.93	> 160	64.7	> 128
$(ABD)_2$-$(EEG)_6$-S12-(D)Pex	1.25	> 160	> 160	> 128	> 128
*HE50 is defined as treatment concentration that leads to 50 % hemolysis.					

Table S1. Selectivity indices (IC50/MIC and HE50/MIC) of ABD-AMP conjugates.

Substrate	Sequence	Proteases known to cleave
S1	(QSY21)–GPLGVRGKLVPRG–K(Cy5)–kkk–CONH2	MMPs, Thrombin
S2	(QSY21)–GPLG–(4-iodo-Phe)–GAR–K(Cy5)–kkk–CONH2	MMP-9
S3	(QSY21)–GRSLSRLTA–K(Cy5)–kkk–CONH2	MMPs
S4	(QSY21)–GLGRG–K(Cy5)–kkk–CONH2	PA Imelysin
S5	(QSY21)–GAGLG–K(Cy5)–kkk–CONH2	PA LasB
S6	(QSY21)–G-Nle(O-Bzl)–Met(O)2–Oic–Abu–G–K(Cy5)–kkk–CONH2	Neutrophil elastase
S7	(QSY21)–GLVPRG–K(Cy5)–kkk–CONH2	Thrombin
S8	(QSY21)–GPLGVRGK–K(Cy5)–kkk–CONH2	MMPs
S9	(QSY21)–GP-(Cha)-G-Cys(Me)-HAG-K(Cy5)–kkk–CONH2	MMPs
S10	(QSY21)–GPVPLSLVMG–K(Cy5)–kkk–CONH2	MMPs, Cathepsins, Thrombin
S11	(QSY21)–GPVGLIGG–K(Cy5)–kkk–CONH2	MMPs
S12	(QSY21)–GPLGLRSWG–K(Cy5)–kkk–CONH2	MMPs, Cathepsins, Thrombin
S13	(QSY21)–GVLKG–K(Cy5)–kkk–CONH2	Plasmin
S14	(QSY21)–GPQGIWGQG–K(Cy5)–kkk–CONH2	Cathepsins, Thrombin
S15	(QSY21)–GKPISLISSG–K(Cy5)–kkk–CONH2	MMPs, Cathepsins
S16	(QSY21)–GGGPG–K(Cy5)–kkk–CONH2	Fibroblast-activation protein
S17	(QSY21)-GILSRIVG-K(Cy5)-kkk-CONH2	Cathepsins, Thrombin

4-iodo-Phe: 4-iodo-L-phenylalanine
Nle(O-Bzl): 6-benzyloxy-L-norleucine
Met(O)2: L-methionine sulfone
Oic: octahydroindole-2-carboxylic Acid
Abu: L-2-aminobutyric acid
Cha: 3-cyclohexyl-L-alanine
Cys(Me): S-methyl-L-cysteine
Small letters denote D-amino acids.

Table S2. List of peptide substrates and sequences.

	**AUC (μM*h)**	**Fold difference in AUC relative to (D)Pex-Cy7**	**$t_{1/2,\alpha}$ (h)**	**$t_{1/2,\beta}$ (h)**
(D)Pex-Cy7	2.08	1	0.589	0.593
ABD-(D)Pex-Cy7	32.9	15.8	0.0879	2.76
ABD-(EEG)$_6$-S12-(D)Pex-Cy7	83.8	40.3	0.585	5.29
(ABD)$_2$-(EEG)$_6$-S12-(D)Pex-Cy7	189	90.8	0.805	11.4
(ZHER2)$_2$-(EEG)$_6$-S12-(D)Pex-Cy7	0.949	0.949	0.0671	0.625

Table S3. Pharmacokinetics evaluation of ABD-AMP conjugates.

	(D)Pex-Cy7	$(ABD)_2$-$(EEG)_6$-S12-(D)Pex-Cy7	Fold difference relative to (D)Pex-Cy7 group
**Lungs**			
AUC ((D)Pex-Cy7)	5.29	13.6	2.58
AUC (Total)		111	21
**Kidneys**			
AUC ((D)Pex-Cy7)	14.1	11.8	0.837
AUC (Total)		137.5	9.73
**Liver**			
AUC ((D)Pex-Cy7)	112	31.2	0.277
AUC (Total)		250	2.22
**Spleen**			
AUC ((D)Pex-Cy7)	23.5	14.3	0.611
AUC (Total)		37.1	1.58
AUC unit in % ID*h/g			

Table S4. AUC table of longitudinal biodistribution study in PAO1-infected mice.

AMP	Sequence (N→C)
(D)Pexiganan	GiGkflkkakkfGkafvkilkk–CONH2
(D)CAMEL0	kwklfkkiGavlkvl–CONH2
Tachyplesin I	KWCFRVCYRGICYRRCR–CONH2, Disulfide bond: C3&C16 and C7&C12
(D)KLA	klaklakklaklak–CONH2
(D)Pexiganan-azide	Azidoacetyl–GiGkflkkakkfGkafvkilkk–CONH2
(D)CAMEL0-azide	Azidoacetyl–GkwklfkkiGavlkvl–CONH2
Tachyplesin I-azide	Azidoacetyl–GKWCFRVCYRGICYRRCR–CONH2, Disulfide bond: C4&C17 and C8&C13
(D)Pexiganan-Cy7-azide	Azidoacetyl–GiGkflkkakkfGkafvkilkk–K(Cy7)–CONH2
(D)KLA-Cy7-azide	Azidoacetyl– Gklaklakklaklak–K(Cy7)–CONH2
Tachyplesin I-Cy7-azide	Azidoacetyl–GKWCFRVCYRGICYRRCRG–K(Cy7)–CONH2

Table S5. List of AMPs and sequences.

Conjugate	Sequence	Mw Calculated (Da)	Mw Measured (Da)
ABD-S1-(D)Pex	G-ABD-His6-Spacer-G-S1-GC-(DBCO-Maleimide)-((D)Pexiganan-azide)	11,629.78	11,628.81
ABD-(EEG)$_4$-S1-(D)Pex	G-ABD-His6-Spacer-(EEG)$_4$-S1-GC-(DBCO-Maleimide)-((D)Pexiganan-azide)	12,833.85	12,834.13
ABD-(EEG)$_6$-S1-(D)Pex	G-ABD-His6-Spacer-(EEG)$_6$-S1-GC-(DBCO-Maleimide)-((D)Pexiganan-azide)	13,464.41	13,464.32
(ABD)$_2$-(EEG)$_6$-S12-(D)Pex	G-ABD-(GGGGS)$_3$-ABD-His6-Spacer-(EEG)$_6$-S12-GC-(DBCO-Maleimide)-((D)Pexiganan-azide)	19,068.54	19,069.17
ABD-(EEG)$_6$-S1-(D)CAMEL0	G-ABD-His6-Spacer-(EEG)$_6$-G-S1-GC-(DBCO-Maleimide)-((D)CAMEL0-azide)	12,757.61	12,758.12
ABD-(EEG)$_6$-S1-Tachyplesin I	G-ABD-His6-Spacer-(EEG)$_6$-G-S1-GC-(DBCO-Maleimide)-(Tachyplesin I-azide)	13,308.11	13,310.81
(ABD)$_2$-(EEG)$_6$-S12-(D)KLA	G-ABD-(GGGGS)$_3$-ABD-His6-Spacer-(EEG)$_6$-S12-GC-(DBCO-Maleimide)-((D)KLA-azide)	18,171.44	18,171.59
ABD-S1-(D)Pex-Cy7	G-ABD-His6-Spacer-G-S1-GC-(DBCO-Maleimide)-((D)Pexiganan-Cy7-azide)	12,422.78	12,424.69
ABD-(EEG)$_4$-S1-(D)Pex-Cy7	G-ABD-His6-Spacer-(EEG)$_4$-G-S1-GC-(DBCO-Maleimide)-((D)Pexiganan-Cy7-azide)	13,626.85	13,626.58
ABD-(EEG)$_6$-S1-(D)Pex-Cy7	G-ABD-His6-Spacer-(EEG)$_6$-G-S1-GC-(DBCO-Maleimide)-((D)Pexiganan-Cy7-azide)	14,257.41	14,259.34
ABD-(EEG)$_8$-S1-(D)Pex-Cy7	GS-His6-SG-ABD-Spacer-(EEG)$_8$-S1-GC-(DBCO-Maleimide)-((D)Pexiganan-Cy7-azide)	15,119.18	15,118.09
ABD-(EEG)$_{10}$-S1-(D)Pex-Cy7	GS-His6-SG-ABD-(EEG)$_{10}$-S1-GC-(DBCO-Maleimide)-((D)Pexiganan-Cy7-azide)	14,418.31	14,417.45
ABD-(EEG)$_6$-S9-(D)Pex-Cy7	G-ABD-His6-Spacer-(EEG)$_6$-S9-GC-(DBCO-Maleimide)-((D)Pexiganan-Cy7-azide)	13,773.8	13,773.31
ABD-(EEG)$_6$-S10-(D)Pex-Cy7	G-ABD-His6-Spacer-(EEG)$_6$-S10-GC-(DBCO-Maleimide)-((D)Pexiganan-Cy7-azide)	13,921.04	13,922.21
ABD-(EEG)$_6$-S11-(D)Pex-Cy7	G-ABD-His6-Spacer-(EEG)$_6$-S11-GC-(DBCO-Maleimide)-((D)Pexiganan-Cy7-azide)	13,563.57	13,564.36
ABD-(EEG)$_6$-S12-(D)Pex-Cy7	G-ABD-His6-Spacer-(EEG)$_6$-S12-GC-(DBCO-Maleimide)-((D)Pexiganan-Cy7-azide)	13,893.91	13,892.14
ABD-(EEG)$_6$-S14-(D)Pex-Cy7	G-ABD-His6-Spacer-(EEG)$_6$-S14-GC-(DBCO-Maleimide)-((D)Pexiganan-Cy7-azide)	13,850.8	13,849.43
ABD-(EEG)$_6$-S15-(D)Pex-Cy7	G-ABD-His6-Spacer-(EEG)$_6$-S15-GC-(DBCO-Maleimide)-((D)Pexiganan-Cy7-azide)	13,909.95	13,909.67
ABD-(EEG)$_6$-S17-(D)Pex-Cy7	G-ABD-His6-Spacer-(EEG)$_6$-S17-GC-(DBCO-Maleimide)-((D)Pexiganan-Cy7-azide)	13,765.82	13,765.54
(ABD)$_2$-(EEG)$_6$-S12-(D)Pex-Cy7	G-ABD-(GGGGS)$_3$-ABD-His6-Spacer-(EEG)$_6$-S12-GC-(DBCO-Maleimide)-((D)Pexiganan-Cy7-azide)	19,861.54	19,860.93

ABD-$(EEG)_6$-NC-(D)Pex-Cy7	G-ABD-His6-Spacer-$(EEG)_6$-C-(DBCO-Maleimide)-((D)Pexiganan-Cy7-azide)	13,026.9	13,026.09
ABD-S1-(D)KLA-Cy7	G-ABD-His6-Spacer-G-S1-GC-(DBCO-Maleimide)-((D)KLA-Cy7-azide)	11,525.68	11,525.48
ABD-$(EEG)_4$-S1-(D)KLA-Cy7	G-ABD-His6-Spacer-$(EEG)_4$-G-S1-GC-(DBCO-Maleimide)-((D)KLA-Cy7-azide)	12,729.75	12,731.09
ABD-$(EEG)_6$-S1-(D)KLA-Cy7	G-ABD-His6-Spacer-$(EEG)_6$-G-S1-GC-(DBCO-Maleimide)-((D)KLA-Cy7-azide)	13,360.31	13,361.28
$(ABD)_2$-$(EEG)_6$-S12-(D)KLA-Cy7	G-ABD-$(GGGGS)_3$-ABD-His6-Spacer-$(EEG)_6$-S12-GC-(DBCO-Maleimide)-((D)KLA-Cy7-azide)	18,964.44	18,965.17
ABD-S1-Tachyplesin I-Cy7	G-ABD-His6-Spacer-G-S1-GC-(DBCO-Maleimide)-(Tachyplesin I -Cy7-azide)	12,323.48	12,324.81
ABD-$(EEG)_4$-S1-Tachyplesin I-Cy7	G-ABD-His6-Spacer-$(EEG)_4$-G-S1-GC-(DBCO-Maleimide)-(Tachyplesin I -Cy7-azide)	13,527.55	13,528.59
ABD-$(EEG)_6$-S1-Tachyplesin I-Cy7	G-ABD-His6-Spacer-$(EEG)_6$-G-S1-GC-(DBCO-Maleimide)-(Tachyplesin I-Cy7-azide)	14,158.11	14,159.6
$(ZHER2)_2$-$(EEG)_6$-S12-(D)Pex-Cy7	G-ZHER2-GGGS-His6-GGGS-ZHER2-His6-Spacer-$(EEG)_6$-S12-GC-(DBCO-Maleimide)-((D)Pexiganan-Cy7-azide)	23,586.44	23,583.4
ABD-$(EEG)_6$-S12-Biotin	G-ABD-His6-Spacer-(EEG)6-S12-GC-(Biotin-Maleimide)	10,564.5	10,565.56
$(ABD)_2$-$(EEG)_6$-S12-Biotin	G-ABD-$(GGGGS)_3$-ABD-His6-Spacer-$(EEG)_6$-S12-GC-(Biotin-Maleimide)	16,532.13	16,533.94
**Component**			
ABD (ABDcon[2])	LKEAKEKAIEELKKAGITSDYYFDLINKA KTVEGVNALKDEILKA		
His6	HHHHHH		
Spacer	SPSTPPTPSPSTPP		
ZHER2 (Z_{HER2}:342[1])	VDNKFNKEMRNAYWEIALLPNLNNQQKR AFIRSLYDDPSQSANLLAEAKKLNDAQA PK		

Table S6. List of ABD-AMP conjugates, sequences, and molecular weights.

Supporting References

(1) Orlova, A.; Magnusson, M.; Eriksson, T. L. J.; Nilsson, M.; Larsson, B.; Höidén-Guthenberg, I.; Widström, C.; Carlsson, J.; Tolmachev, V.; Ståhl, S.; Nilsson, F. Y. Tumor Imaging Using a Picomolar Affinity HER2 Binding Affibody Molecule. *Cancer Res.* **2006**, *66* (8), 4339–4348. https://doi.org/10.1158/0008-5472.CAN-05-3521.

(2) Jacobs, S. A.; Gibbs, A. C.; Conk, M.; Yi, F.; Maguire, D.; Kane, C.; O'Neil, K. T. Fusion to a Highly Stable Consensus Albumin Binding Domain Allows for Tunable Pharmacokinetics. *Protein Eng. Des. Sel.* **2015**, *28* (10), 385–393. https://doi.org/10.1093/protein/gzv040.
